# Both α2,3- and α2,6-Linked Sialic Acids on O-Linked Glycoproteins Act as Functional Receptors for Porcine Sapovirus

**DOI:** 10.1371/journal.ppat.1004172

**Published:** 2014-06-05

**Authors:** Deok-Song Kim, Myra Hosmillo, Mia Madel Alfajaro, Ji-Yun Kim, Jun-Gyu Park, Kyu-Yeol Son, Eun-Hye Ryu, Frederic Sorgeloos, Hyung-Jun Kwon, Su-Jin Park, Woo Song Lee, Duck Cho, Joseph Kwon, Jong-Soon Choi, Mun-Il Kang, Ian Goodfellow, Kyoung-Oh Cho

**Affiliations:** 1 Laboratory of Veterinary Pathology, College of Veterinary Medicine, Chonnam National University, Gwangju, Republic of Korea; 2 Division of Virology, Department of Pathology, University of Cambridge, Cambridge, United Kingdom; 3 Bioindustry Research Center, Korea Research Institute of Bioscience and Biotechnology, Jeongeup, Republic of Korea; 4 Department of Laboratory Medicine, Chonnam National University Hwasun Hospital, Jeollanam-do, Republic of Korea; 5 Division of Life Science, Korea Basic Science Institute, Daejeon, Republic of Korea; The Scripps Research Institute, United States of America

## Abstract

Sapovirus, a member of the *Caliciviridae* family, is an important cause of acute gastroenteritis in humans and pigs. Currently, the porcine sapovirus (PSaV) Cowden strain remains the only cultivable member of the *Sapovirus* genus. While some caliciviruses are known to utilize carbohydrate receptors for entry and infection, a functional receptor for sapovirus is unknown. To characterize the functional receptor of the Cowden strain of PSaV, we undertook a comprehensive series of protein-ligand biochemical assays in mock and PSaV-infected cell culture and/or piglet intestinal tissue sections. PSaV revealed neither hemagglutination activity with red blood cells from any species nor binding activity to synthetic histo-blood group antigens, indicating that PSaV does not use histo-blood group antigens as receptors. Attachment and infection of PSaV were markedly blocked by sialic acid and *Vibrio cholerae* neuraminidase (NA), suggesting a role for α2,3-linked, α2,6-linked or α2,8-linked sialic acid in virus attachment. However, viral attachment and infection were only partially inhibited by treatment of cells with sialidase S (SS) or *Maackia amurensis* lectin (MAL), both specific for α2,3-linked sialic acid, or *Sambucus nigra* lectin (SNL), specific for α2,6-linked sialic acid. These results indicated that PSaV recognizes both α2,3- and α2,6-linked sialic acids for viral attachment and infection. Treatment of cells with proteases or with benzyl 4-O-β-D-galactopyranosyl-β-D-glucopyranoside (benzylGalNAc), which inhibits *O*-linked glycosylation, also reduced virus binding and infection, whereas inhibition of glycolipd synthesis or *N*-linked glycosylation had no such effect on virus binding or infection. These data suggest PSaV binds to cellular receptors that consist of α2,3- and α2,6-linked sialic acids on glycoproteins attached via *O*-linked glycosylation.

## Author Summary

Although enteropathogenic sapoviruses and noroviruses are leading causes of acute gastroenteritis in both humans and animals, the study of viral pathogenesis and immunity of these ubiquitous pathogens has been hampered due to the lack of a fully permissive cell culture system. Porcine sapovirus Cowden strain provides a suitable system that can be used to identify the molecular mechanisms of viral pathogenesis. Previous studies have shown that carbohydrates and glycolipids play important roles in the attachment of members of the *Caliciviridae*; histo-blood group antigens (HBGAs) are used by *Norovirus* genogroups I to IV, as well as members of the *Lagovirus*, and *Recovirus* genera, whereas terminal sialic acid is recognized as a receptor for feline calicivirus and murine norovirus. To date, however, the role of carbohydrates in the life cycle of sapoviruses has remained largely unknown. We found that porcine sapovirus binds to susceptible host cells through both α2,3- and α2,6-linked terminal sialic acids which are attached to *O*-linked glycoproteins. These efforts, findings and insights will significantly contribute to a better understanding of the sapovirus life cycle.

## Introduction

Caliciviruses (family *Caliciviridae*) are small (27–40 nm), non-enveloped, icosahedral viruses that possess a single-strand, plus-sense genomic RNA of 7–8 kb [1]. Caliciviruses are important veterinary and human pathogens which are associated with a broad spectrum of diseases in their respective hosts. A member of the genus *Lagovirus*, rabbit hemorrhagic disease virus (RHDV), is associated with a fatal liver disease in rabbits [2]. Feline calicivirus (FCV), a member of the genus *Vesivirus*, causes respiratory disease in cats [3], [4]. Caliciviruses in the genera *Norovirus* and *Sapovirus* are important acute gastroenteritis pathogens in humans and animals [5], [6]. Each year, human noroviruses cause at least 1.1 million episodes and 218,000 deaths in developing nations as well as approximately 900,000 cases of pediatric gastroenteritis in industrialized nations [7]. Sapoviruses have also been associated with gastroenteritis outbreaks and with disease in pediatric patients [1]. The genus *Sapovirus* can be divided into five genogroups (GI–GV), among which GI, GII, GIV and GV are known to infect humans, whereas GIII infects porcine species [8]. No fully permissive cell culture system currently exists for the enteric caliciviruses associated with gastroenteritis in humans, hampering the study of viral pathogenesis and immunity of these ubiquitous pathogens [1].

The initial events in a viral infection are induced by binding of the virus to the surface of the host cell, followed by penetration or release of the virus particle into the cytoplasm of the cell. Binding occurs through interactions between the virion and receptors on the plasma membrane of the target cell, and consequently receptors are important determinants of viral tissue tropism and pathogenesis [1]. Among the members of the *Calicivirdae* family, an attachment factor for RHDV was identified as H-type 2 histo-blood group antigen (HBGA), and this led to further studies identifying factors involved in the attachment of the other members of the family [9]. HBGAs function as the attachment factor of both human and bovine noroviruses [5], [10], while sialic acid linked with gangliosides acts as at least part of the murine norovirus (MNV) receptor [11]. In addition, Tulane virus, the newly discovered rhesus monkey calicivirus, uses HBGA as a receptor [12]. FCV is reported to recognize terminal sialic acid on an *N*-linked glycoprotein for attachment [4], with junctional adhesion molecule-1 (JAM-1) functioning as a receptor to facilitate FCV penetration and infection of host cells [5], [13]. Although these reports strongly suggest that the recognition of a carbohydrate receptor may be a common feature of many caliciviruses, it is also clear that different caliciviruses recognize different carbohydrate receptors [5]. Importantly, however, virus-like particles of human sapovirus genogroups GI and GV strains are known not to bind salivary HBGAs or synthetic carbohydrates [14].

The close genetic relationship of noroviruses and sapoviruses found in humans and animals has led to concern over the possibility of zoonotic transmission of these viruses [15]. Although animal sapoviruses and noroviruses have not yet been isolated from humans, the detection of antibodies against bovine GIII norovirus and canine GVI norovirus in human serum samples [16], [17], and the detection of human-like GII.4 norovirus in porcine and bovine fecal samples [18] suggest the possible zoonotic transmission of noroviruses. Furthermore, it has been demonstrated that human norovirus GII4-HS66 strain can induce diarrhea and intestinal lesions in the proximal small intestine of gnotobiotic piglets or calves [19]. There is also evidence to suggest that highly conserved receptors across host species can be shared by different viruses, even among different genera or families [10], [20], [21]. Such concerns have prompted us to investigate the ability of porcine sapovirus (PSaV) to recognize carbohydrates present on cultured cells of porcine origin and small intestinal epithelial cells of piglets. In this study, we demonstrate that PSaV binds to both α2,3- and α2,6-linked sialic acids present on an *O*-linked glycoprotein.

## Results

### Attachment and infection of PSaV requires carbohydrate moieties

**Figure 1:**
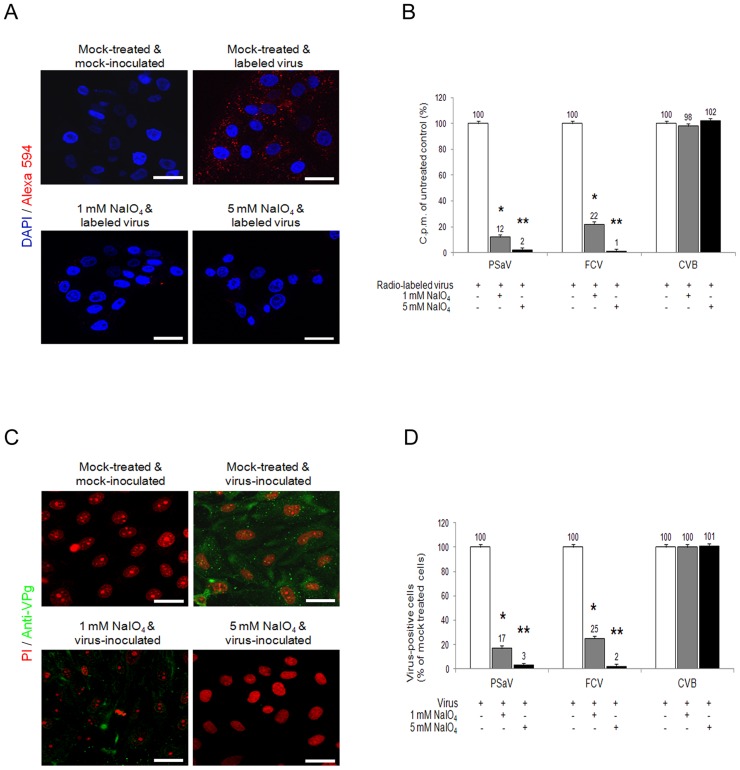
Porcine sapovirus Cowden strain binding and infection requires carbohydrate moieties. LLC-PK cells were pretreated with 1 mM or 5 mM of NaIO_4_ to remove carbohydrate moieties. (A) Alexa 594 or Alexa 594-labeled PSaV (MOI of 100 pfu/cell) were bound to pretreated LLC-PK cells, and were subsequently examined for bound virus by confocal microscopy. (B) ^35^[S]Methionine/Cysteine-labeled PSaV, control FCV or CVB3 (50,000 c.p.m.) were bound to LLC-PK, CRFK or HeLa cells which were pretreated with or without NaIO_4_. Binding of radio-labeled PSaV, FCV or CVB3 was measured by liquid scintillation counting. (C) PSaV (MOI of 0.1 pfu/cell) was inoculated to NaIO_4_ pretreated LLC-PK cells, and was subsequently analyzed by immunofluorescence assay to detect the viral antigen VPg, using a rabbit polyclonal antibody 72 h post infection. (D) PSaV, FCV or CVB3 positive cells (%) were quantified in three independent microscope fields. All experiments were performed three independent times, and figures A and C show one representative set of results. The scale bars correspond to 20 µm. Error bars indicate SD from triplicate samples. *p<0.05, **p<0.005.

Although carbohydrate moieties are known to act as receptors or attachment factors for various caliciviruses [5], their role as receptors or attachment factors for members of the *Sapovirus* genus remains unknown. To determine if PSaV Cowden strain requires carbohydrate moieties for binding and infection, we removed the carbohydrate moieties from permissive porcine LLC-PK cells by treatment with sodium periodate (NaIO_4_), which is known to cleave carbohydrate groups without altering proteins or membranes [4], [22], [23]. Pretreatment of LLC-PK cells with 1 mM or 5 mM NaIO_4_ markedly reduced the binding of Alexa 594-labeled PSaV Cowden strain compared to mock treated control (Fig. 1A). To quantify the effect of NaIO_4_ treatment more accurately, LLC-PK cells were pretreated in a similar manner, and were incubated with radio-labeled PSaV Cowden strain. Cells were washed thoroughly, and virus binding was determined by liquid scintillation counting. Binding of PSaV Cowden strain was reduced to 12% of the levels observed in mock treated cells with 1 mM NaIO_4_, and to 2% in cells treated with 5 mM NaIO_4_ (Fig. 1B). The infection rate, as determined by staining cells for the viral antigen VPg, was also significantly reduced; infection rates of 17% and 3% were observed for 1 mM and 5 mM NaIO_4_, respectively, when compared with mock-treated cells (Fig.1C and 1D). A similar degree of inhibition of binding and infection was observed in FCV F9 strain-infected Crandall-Reese feline kidney (CRFK) cells that were pretreated with NaIO_4_ (Fig.1B and 1D). However, binding and infection of coxsackievirus B3 (CVB3) Nancy strain, which is known to us decay-accelerating factor as a receptor [24], was not influenced by the pretreatment of HeLa cells with 1 mM or 5 mM NaIO_4_ (Fig. 1B and 1D, and Fig. S1). In addition, pretreatment of LLC-PK cells with 5 mM NaIO_4_ had no effect on binding of MNV-1 strain CW1 or vesicular stomatitis virus glycoprotein (VSV-G protein) pseudotyped lentiviruses (Fig. S1). These data strongly indicated that like FCV F9 strain, PSaV Cowden strain utilizes carbohydrate moieties for binding and infection.

### PSaV does not agglutinate any red blood cell (RBC) from various species

**Figure 2:**
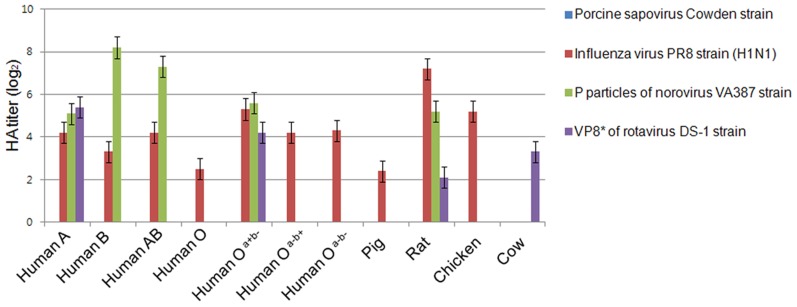
Porcine sapovirus Cowden strain does not agglutinate RBCs from various species. Agglutination of pig, cow, chicken, rat and human (ABO and Lewis types) RBCs by PSaV Cowden strain, human influenza A virus strain A/Puerto Rico/8/34 (H1N1) (PR8 virus), P particles of norovirus VA387 strain, or VP8* of human rotavirus DS-1 strain were determined by hemagglutination assay. Results are shown as average hemagglutination titers (Log_2_) obtained from triplicate samples in three independent experiments. Error bars represent mean ± SD.

HBGAs are complex carbohydrates present on the surfaces of RBCs, and mucosal epithelia, mucin of respiratory, genitourinary, and digestive tract [25]. Among caliciviruses, human and bovine noroviruses, RHDV, and Tulane virus are known to use HBGAs as receptors, resulting in the agglutination of RBCs [5], [12]. To determine if PSaV Cowden strain agglutinated RBCs, hemagglutination assay (HA) was performed by using RBCs that originated from various animal species including pigs, rats, chickens, and humans, which was further classified into ABO and Lewis types. PSaV Cowden strain displayed no hemagglutination activity with RBCs from any species at 4°C or 20°C incubation (Fig. 2). In contrast, influenza A virus PR8 (H1N1) strain agglutinated human, rat, pig and chicken RBCs. P particles of human norovirus VA387 strain and a VP8* of human rotavirus DS-1 strain confirmed binding to corresponding HBGAs [26] (Fig. 2). Whilst positive controls showed differing degrees of HA activities against RBCs from various species, the absence of HA activity by PSaV indicated that PSaV Cowden strain may not recognize HBGAs as receptors for its binding and infection of host cells.

### PSaV does not interact with synthetic HBGAs

**Figure 3:**
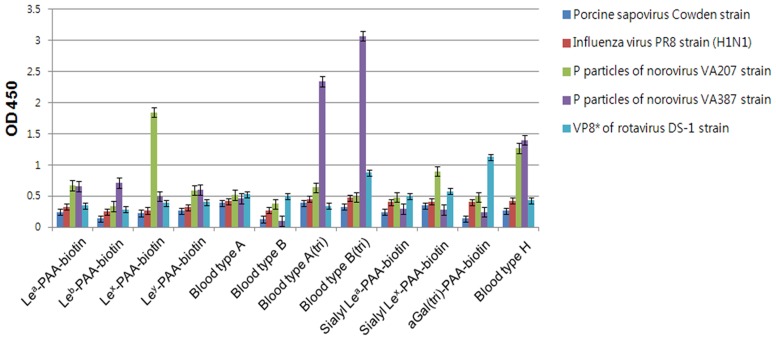
Porcine sapovirus Cowden strain does not bind synthetic histo-blood group antigens (HBGAs). Binding of synthetic HBGAs by PSaV Cowden strain, P particles of norovirus VA387 and VA207 strains, human influenza A virus strain A/Puerto Rico/8/34 (H1N1) (PR8 virus), and VP8* of human rotavirus DS-1 strain were determined using HRP-conjugated-streptavidin. The signal intensities were visualized by TMB at 450 nm in three independent experiments. Error bars represent mean ± SD.

In order to confirm whether PSaV recognizes HBGAs as receptors, a synthetic HBGA binding assay was conducted [26], [27]. The descriptions and structures of all the oligosaccharides tested are provided in Table 1. Consistent with the HA assay, PSaV Cowden strain did not bind to immobilized synthetic oligosaccharides, including the A and H types, both of which are known to be expressed in pigs (Fig. 3) [28]. However, recombinant proteins consisting of the P particles of human norovirus VA387 bound to synthetic oligosaccharides of A type, B type and H type, the P particles of human norovirus VA207 bound to synthetic oligosaccharides Lewis types and H type, and a VP8* of human rotavirus DS-1 bound to synthetic oligosaccharides B type and αGal, respectively (Fig. 3) [5], [26]. Collectively, these results demonstrated that PSaV Cowden strain does not utilize HBGAs as receptors.

**Table 1:** Synthetic oligosaccharide used in this study.

Name	Identification No.		Structure
Le^a^-PAA-biotin		1	Fucα1-2Galβ1-3(Fucα1-4)GlcNAcβ1-PAA-biotin
Le^b^-PAA-biotin		2	Galβ1-3(Fucα1-4)GlcNAcβ1-PAA-biotin
Le^x^-PAA-biotin		3	Galβ1-4(Fucα1-3)GlcNAcβ1-PAA-biotin
Le^y^-PAA-biotin		4	Fucα1-2Galβ1-4(Fucα1-3)GlcNAcβ1-PAA-biotin
Blood type A		5	GalNAcα1-3Galβ-PAA-biotin
Blood type B		6	Galα1-3Galβ-PAA-biotin
Blood type A. (tri)-PAA-biotin		7	GalNAcα1-3(Fucα1-2)Galβ1-PAA-biotin
Blood type B. (tri)-PAA-biotin		8	Galα1-3(Fucα1-2)Galβ-PAA-biotin
Sialyl Le^a^-PAA-biotin		9	NeuAcα2-3Galβ(Fucα1-4)1-3GlcNAcβ1-PAA-biotin
Sialyl Le^x^-PAA-biotin		10	NeuAcα2-3Galβ(Fucα1-3)1-4GlcNAcβ1-PAA-biotin
αGal. (tri)-PAA-biotin		11	Galα1-3Galβ1-4GlcNAcb-PAA-biotin, sp = -NHCOCH2NH-
Blood type H		12	Fucα1-2Galβ-PAA-biotin

### Sialic acid acts as a functional receptor for PSaV

**Figure 4:**
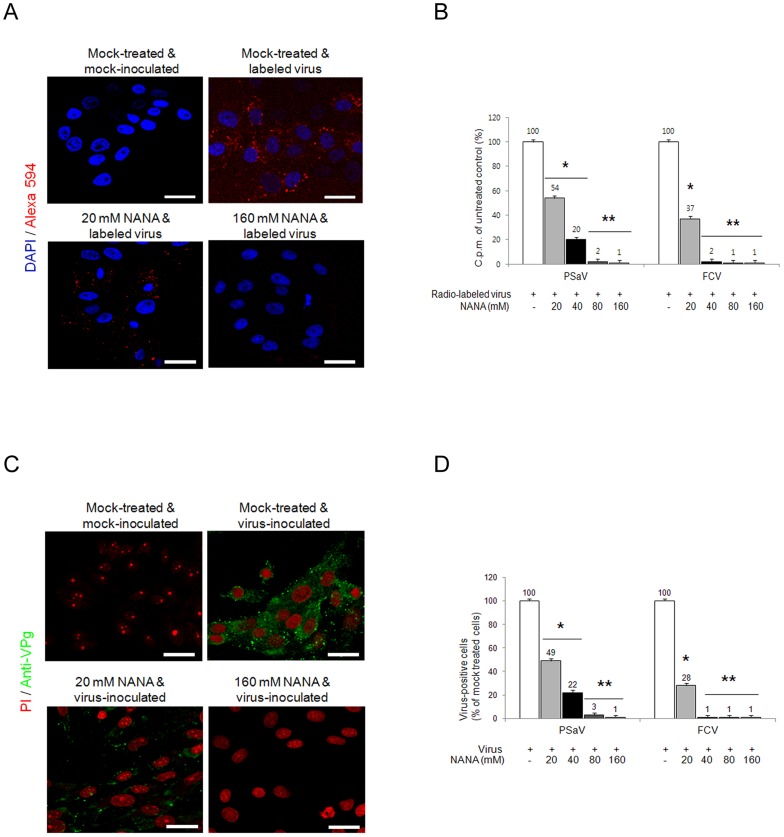
Porcine sapovirus Cowden strain binding and infection is blocked by *N*-acetyl neuraminic acid (NANA). PSaV or control FCV were pre-incubated with a soluble form of NANA at the indicated concentrations to block sialic acid receptors. (A) Alexa 594-labeled PSaV (MOI of 100 pfu/cell) was bound to pretreated LLC-PK cells, and was analyzed for virus binding activity by confocal microscopy. (B) ^35^[S]Methionine/Cysteine-labeled PSaV or control FCV (50,000 c.p.m.) were bound to pretreated LLC-PK or CRFK cells, and binding of radio-labeled PSaV or FCV was measured by liquid scintillation counting. (C) PSaV (MOI of 0.1 pfu/cell) was incubated with pretreated LLC-PK cells, and was subsequently analyzed by immunofluorescence assay with rabbit polyclonal antibody against PSaV VPg protein 72 h post infection. (D) PSaV or FCV positive cells (%) were enumerated in three independent microscope fields. All experiments were performed in triplicate and figures A and C show representative sets of results. The scale bars correspond to 20 µm. Error bars represent mean ± SD. *p<0.05, **p<0.005.

Sialic acid is an abundant carbohydrate moiety on the cell surface [25], which acts as a functional receptor for many viruses, including caliciviruses [4], [11]. To determine if sialic acid is a functional receptor for the PSaV, PSaV Cowden strain was incubated with various concentrations (20–160 mM) of the sialic acid containing molecule, *N*-acetyl neuraminic acid (NANA), and was then inoculated with LLC-PK cells. NANA at 20 mM significantly reduced the binding activity of Alexa 594-labeled PSaV Cowden strain, and almost completely inhibited binding at 80 mM (Fig. 4A). Binding of radio-labeled PSaV Cowden strain or FCV F9 strain was reduced by NANA in a dose-dependent manner, and was almost completely abolished at 80 mM NANA for PSaV Cowden strain and 40 mM NANA for FCV F9 strain (Fig. 4B). Infection of cells with PSaV Cowden strain was also reduced by incubation with NANA in a dose-dependent manner, and was almost completely inhibited at 80 mM NANA (Fig. 4C and 4D). Similar observations were found with FCV at 40 mM NANA (Fig. 4D). In addition, when plaque reduction assays were performed, PSaV infection was blocked at 80 mM NANA (Fig. S2). Among other monosaccharides and oligosaccharides, *N*-glycolyl neuraminic acid also inhibited PSaV binding and infection of LLC-PK cells in a dose-dependent manner, but no inhibitory effect of galactose or sialyllactose was found regardless of the concentration (data not shown).

**Figure 5:**
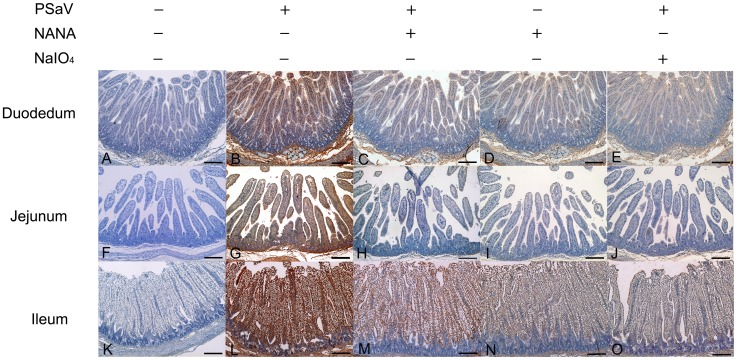
Attachment of porcine sapovirus Cowden strain to porcine intestinal sections is blocked by *N*-acetyl neuraminic acid (NANA) or pretreatment with NaIO_4_. The ability of PSaV to bind to porcine intestinal tissue sections from the duodenum (A–E), jejunum (F–J), and ileum (K–O) was analyzed by immunohistochemistry as described in the Materials and Methods section. In some cases, sections were pretreated with 10 mM NaIO_4_ prior to the addition of virus (E,J,O) or virus samples were premixed with 160 mM NANA prior to incubating with tissue sections (C,H,M). Binding of PSaV to cells was identified using immunohistochemistry and positive binding is indicated by a red/brown color. Scale bars correspond to 200 µm. This experiment was repeated three independent times and one representative set of results is shown.

PSaV infects small intestinal epithelial cells, leading to villous atrophy [29]. To confirm whether PSaV binding in small intestinal epithelial cells is also dependent on sialic acid and blocked by NANA, PSaV was incubated with 160 mM of NANA, and was then incubated with porcine small intestinal tissue sections. Duodenal, jejunal and ileal tissue sections incubated with PSaV Cowden strain alone showed a positive signal for PSaV antigens on the villous epithelial cells. The very weak signal observed in the presence of NANA alone is seen only in the duodenal and ileal tissue sections, probably due to increased non-specific binding of the anti-PSaV antibody (Fig. 5). In contrast to these results, intestinal tissue sections incubated with a mixture of PSaV and 160 mM NANA showed markedly decrease of PSaV antigen intensity (Fig. 5). In addition, pretreatment of small intestinal sections with 1 mM NaIO_4_ (data not shown) or 10 mM NaIO_4_ markedly reduced the binding activity of PSaV Cowden strain (Fig. 5). To rule out any non-specific effects of NANA addition on the integrity of carbohydrate moieties on the tissue sections the effect of NANA pre-incubation on binding of the P domain of human norovirus VA387, which recognizes HBGAs but not sialic acids as attachment factor, was examined [30]. As expected, a mixture of P domain of VA387 strain and 160 mM NANA had no influence on binding to intestinal epithelial cells (Fig. S3). Moreover, binding of the P domain to intestinal epithelial cells was markedly reduced by the pretreatment of 10 mM NaIO_4_ but not by 1 mM NaIO_4_ (Fig. S3). These results fit with previous observations that 1 mM NaIO_4_ eliminates terminal sialic acids only but 10 mM NaIO_4_ removes terminal sialic acids as well as HBGAs on carbohydrate moieties [10]. Taken all together, these data strongly indicated that PSaV binds to sialic acid on the cell surface.

### Both α2,3-linked and α2,6-linked sialic acids are used for PSaV attachment and infection

**Figure 6:**
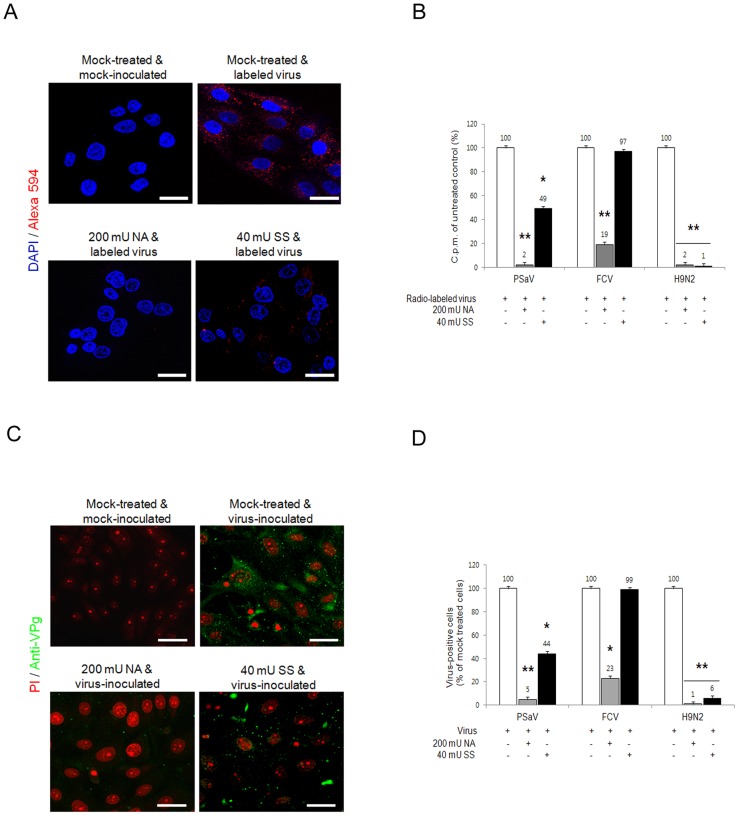
Porcine sapovirus binding and infection requires terminal sialic acids. LLC-PK cells were treated with *V. cholerae* neuraminidase (NA; removes α2,3-, α2,6- and α2,8-linked sialic acid) or sialidase S (SS; removes α2,3-linked sialic acid) from *Streptococcus pneumoniae* at the indicated concentrations. (A) NA- or SS-treated cells were incubated with either Alex 594 alone or Alexa 594-labeled PSaV (MOI of 100 pfu/cell), and bound virus was detected by confocal microscopy. (B) ^35^[S]Methionine/Cysteine-labeled PSaV, FCV or influenza virus (H9N2) (50,000 c.p.m.) were bound to LLC-PK, CRFK or MDCK cells, respectively, pretreated with or without SS indicated, and the binding of radio-labeled PSaV, FCV or influenza virus was quantitated by liquid scintillation counting. (C) PSaV (MOI of 0.1 pfu/cell) was incubated with pretreated LLC-PK cells, and was subsequently analyzed by immunofluorescence assay with rabbit polyclonal antibody against PSaV VPg protein 72 h post infection. (D) PSaV, FCV or influenza virus positive cells (%) were enumerated in three independent microscope fields. All experiments were performed independently three times and figures A and C show a single representative set of results. The scale bars correspond to 20 µm. Error bars denote mean ± SD. *p<0.05, **p<0.005.

Sialic acid is attached to glycans via α2,3-, α2,6- or α2,8-linkages. To determine if PSaV requires these linkages for PSaV binding and infection, LLC-PK cells were pretreated with 200 mU *V. cholerae* neuraminidase (NA) ml^−1^, which cleaves α2,3-linked, α2,6-linked and α2,8-linked sialic acids from the underlying glycans [4]. Treated cells were then incubated with either Alexa 594- or radio-labeled PSaV Cowden strain. Pretreatment with NA markedly reduced the binding of Alexa 594-labeled PSaV (Fig. 6A), and radio-labeled PSaV binding was reduced to 2% of the levels observed in mock treated cells (Fig. 6B). Infection assays, performed by indirect immunofluorescence for the viral antigen VPg, showed almost complete inhibition of PSaV infection at 200 mU NA ml^−1^ (Fig. 6C and 6D). A similar degree of reduction in binding and infection was observed in the cells infected with α2,6-linked sialic acid-dependent FCV F9 strain (Fig. 6B and 6D) [4] and α2,3-linked sialic acid-dependent influenza virus Kr96 strain (H9N2) (Fig. 6B and 6D, and Fig. S4A and S4B) [31] after pretreatment with NA. In addition, pretreatment of NA had no effect on binding of P domain of human norovirus VA387 strain (Fig. S5).

To further identify which specific linkage is used for PSaV binding and infection, LLC-PK cells were initially pretreated with sialidase S (SS) from *Streptococcus pneumoniae*, which exclusively cleaves α2,3-linked sialic acid from complex carbohydrates and glycoproteins [4]. Pretreatment of LLC-PK cells with 40 mU SS ml^−1^ reduced PSaV binding to 49% (Fig. 6A and 6B) and decreased PSaV infection to 44% (Fig. 6C and 6D). Importantly, complete inhibition of binding or infection was not achieved, even with increasing doses of SS (data not shown). As expected, pretreatment of 40 mU SS ml^−1^ significantly blocked binding and infection of α2,3-linked sialic acid-dependent influenza virus Kr96 strain (H9N2) [31] (Fig. 6B and 6D, and Fig. S4A and S4B). In contrast, pretreatment of 40 mU SS ml^−1^ had no effect on α2,6-linked sialic acid-dependent FCV F9 binding or infection (Fig. 6B and 6D) [4] as well as binding of P domain of HBGAs-dependent human norovirus VA387 strain (Fig. S5) [30]. These data suggested that PSaV Cowden strain may use not only α2,3-linked but also α2,6-linked sialic acids.

**Figure 7:**
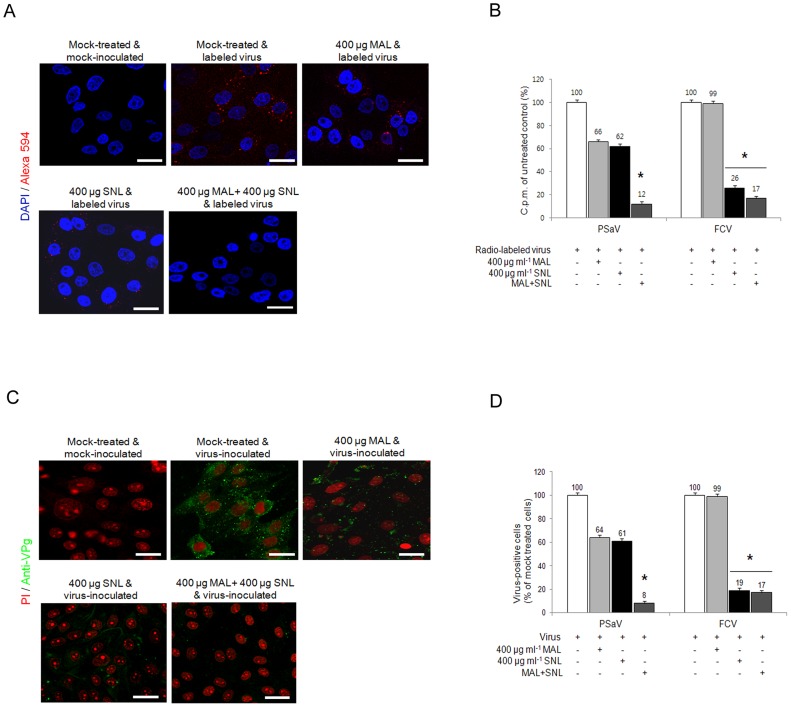
Porcine sapovirus interacts with α2,3- and α2,6-linked sialic acids. LLC-PK cells were pretreated with *M. amurensis* lectin (MAL; preferentially binds to α2,3-sialic acid), *Sambucus nigra* lectin (SNL; preferentially binds to α2,6-linked sialic acid), or combination of both lectins as indicated. (A) Alexa 594 alone or Alexa 594-labeled PSaV (MOI of 100) were bound to pretreated LLC-PK cells, and were observed for their binding activity under confocal microscopy. (B) ^35^[S]Methionine/Cysteine-labeled PSaV or FCV (50,000 c.p.m.) were bound to LLC-PK or CRFK cells which were pretreated with or without lectins, and binding of radio-labeled PSaV or FCV was measured by liquid scintillation counting. (C) Mock or PSaV (MOI of 0.1) were inoculated to pretreated LLC-PK cells, and were analyzed by immunofluorescence assay with rabbit polyclonal antibody against PSaV VPg protein 72 h post infection. (D) PSaV or FCV positive cells (%) were enumerated in three independent microscope fields. All experiments were performed in triplicate and figures A and C show representative sets of results. The scale bars correspond to 20 µm. The error bars represent the mean ± SD. *p<0.05.

In order to further identify the sialic acid linkages required for PSaV attachment and infection, LLC-PK cells were pretreated with specific lectins to block each specific isoform of sialic acid: 1) 400 µg *Maackia amurensis* lectin (MAL) ml^−1^, which binds preferentially to α2,3-linked sialic acid, and 2) 400 µg *Sambucus nigra* lectin (SNL) ml^−1^, which binds preferentially to α2,6-linked sialic acid [4]. Individual pretreatment of both lectins reduced, but did not completely block PSaV binding (Fig. 7A). Quantitation of PSaV binding by using radio-labeled virus also demonstrated reduced binding, with 66% and 62% binding observed, after pretreatment with MAL and SNL, respectively (Fig. 7B). Likewise, infection of LLC-PK cells by PSaV Cowden strain was decreased to 64% by 400 µg MAL ml^−1^ and 61% by 400 µg ml^−1^ SNL (Fig. 7C and 7D). In contrast, MAL had no effect on FCV, but SNL significantly reduced binding and infection (Fig. 7B and 7D). To establish whether PSaV recognizes both α2,3-linked and α2,6-linked sialic acids, LLC-PK cells were pretreated with mixtures of both MAL and SNL, and were then infected with PSaV Cowden strain. PSaV binding and infection were decreased, and complete reduction was observed by the treatment of a mixture at 400 µg MAL ml^−1^ and 400 µg SNL ml^−1^ (Fig. 7A–7D). Taken together, all findings above confirm that PSaV Cowden strain uses both α2,3-linked and α2,6-linked sialic acids for binding and infection.

### Sialic acid for PSaV binding and infection is attached by an *O*-glycosylation to a glycoprotein

**Figure 8:**
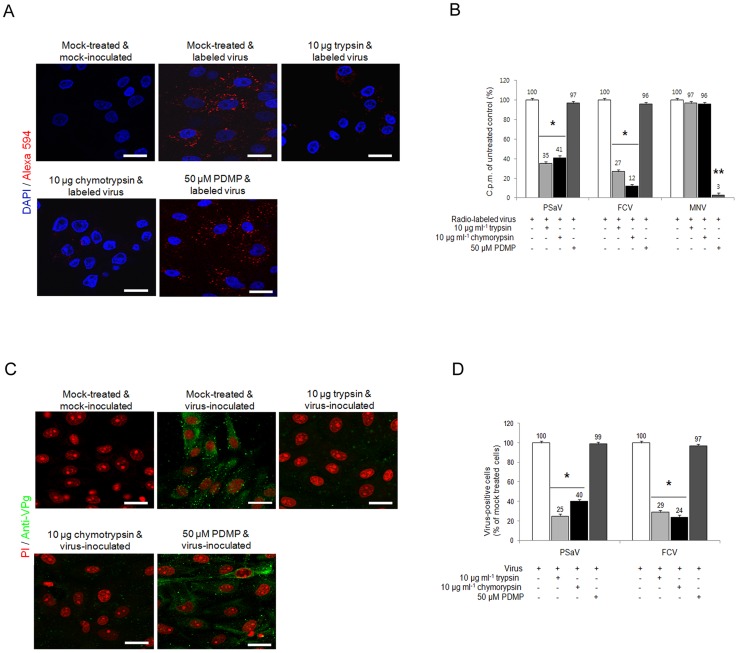
Porcine sapovirus Cowden strain interacts with sialic acid on glycoprotein. LLC-PK cells were pre-incubated with trypsin (protease), chymotrypsin (protease) or PDMP (lipid metabolic inhibitor) at the indicated concentrations to examine which glycan moieties sialic acid is attached to. (A) Alexa 594 alone or Alexa 594-labeled PSaV (MOI of 100) were bound to pretreated LLC-PK cells, and were observed for their binding activity under confocal microscopy. (B) ^35^[S]Methionine/Cysteine-labeled PSaV, FCV or MNV (50,000 c.p.m.) were bound to LLC-PK, CRFK or RAW264.7 cells pretreated with or without proteases or PDMP, and binding of radio-labeled PSaV was measured by liquid scintillation counting. (C) Mock or PSaV (MOI of 0.1) were inoculated to pretreated LLC-PK cells, and were analyzed by immunofluorescence assay with rabbit polyclonal antibody against PSaV VPg protein 72 h post infection. (D) PSaV or FCV positive cells (%) were enumerated in three independent microscope fields. All experiments were performed in triplicate and figures A and C show representative sets of results. The scale bars correspond to 20 µm. Error bars show SD in each sample. *p<0.05.

Sialic acids are typically found at the terminal position of *N*- and *O*-linked glycans attached to the cell surface and to secreted glycoproteins or glycosphingolipids [32]. To test whether sialic acid moieties used for PSaV binding attach to a glycoprotein, LLC-PK cells were pretreated with either trypsin or chymotrypsin, and were then inoculated with Alexa 594-labeled or radio-labeled PSaV Cowden strain. As shown in Fig. 8A and 8B, pretreatment of 10 µg trypsin ml^−1^ and 10 µg chymotrypsin ml^−1^ reduced PSaV attachments to 35% and 41%, respectively, compared to mock treated and PSaV-inoculated control. Furthermore, individual pretreatment of cells with proteases reduced PSaV infection to 25% by trypsin and to 40% by chymotrypsin (Fig. 8C and 8D). Comparatively, FCV binding and infection was reduced by trypsin or chymotrypsin pretreatments, to as low as 12% or 24%, respectively (Fig. 8B and 8D). This inhibition to PSaV binding and infection by trypsin or chymotrypsin pretreatments suggested that like FCV, PSaV Cowden strain binds to sialic acid attached to a glycoprotein.

To investigate if PSaV also utilizes glycolipid containing sialic acid moieties, LLC-PK cells were pretreated with 50 µM DL-*Threo*-1-phenyl-2-decanoylamino-3-morpholino-1-propanol (PDMP), a well-known inhibitor of glucosylceramide synthase, and were then infected with PSaV Cowden strain. Regardless of PDMP pretreatment, PSaV bound to and replicated in cells to levels which were identical to those observed in mock treated cells (Fig. 8A–D). Likewise, FCV F9 strain bound to and replicated in CRFK cells despite pretreatment of cells with 50 µM PDMP (Fig. 8B and 8D). As expected, binding of MNV-1 CW1 strain which uses glycolipid as a receptor [11] was significantly inhibited by pretreatment of 50 µM PDMP (Fig. 8 and Fig. S6 and S7). In addition, binding of a VSV-G protein pseudotyped lentivirus, binding of which is also known to at least partially involve glycolipids [33], was also affected by PDMP treatment of porcine LLC-PK cells (Fig. S7). These data confirm that PDMP treatment of LLC-PK cells reduced glycolipid synthesis, therefore confirming that PSaV Cowden strain attaches and infects cells via a glycoprotein containing sialic acid moieties.

**Figure 9:**
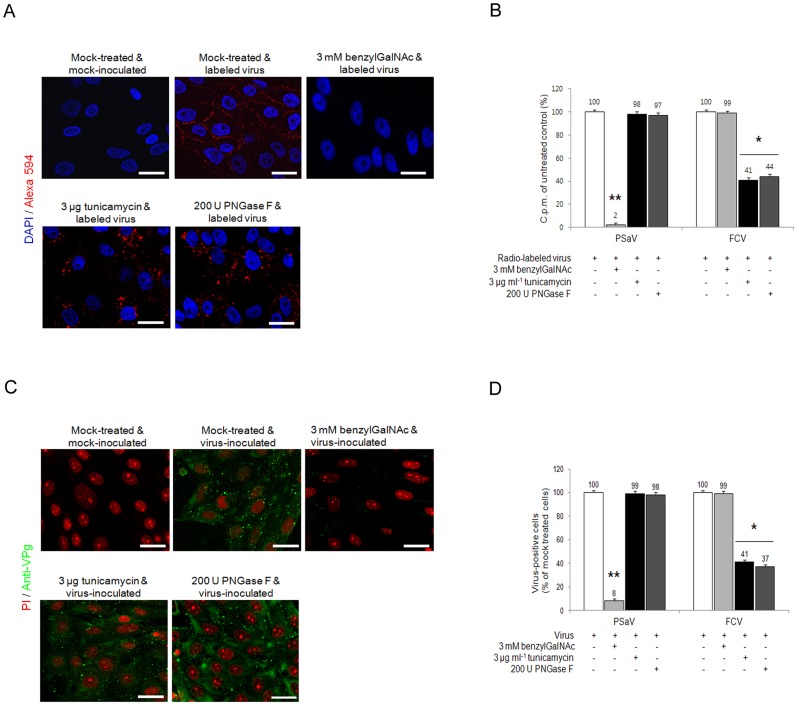
Porcine sapovirus Cowden strain interacts with sialic acid on *O*-linked glycoprotein. LLC-PK cells were pre-incubated with tunicamycin (*N*-linked blocker), PNGase F (*N*-linked blocker) or benzylGalNAc (*O*-linked blocker) at the indicated concentrations to confirm whether sialic acid is linked to an *N* or *O*-glycoprotein. (A) Alexa 594 alone or Alexa 594-labeled PSaV (MOI of 100) were bound to pretreated LLC-PK cells, and were observed for their binding activity under confocal microscopy. (B) ^35^[S]Methionine/Cysteine-labeled PSaV or FCV (50,000 c.p.m.) were bound to LLC-PK or CRFK cells pretreated with or without tunicamycin, PNGase F or benzylGalNAc, and the binding of radio-labeled PSaV was measured by liquid scintillation counting. (C) Mock or PSaV (MOI of 0.1) were inoculated to pretreated LLC-PK cells, and were analyzed by immunofluorescence assay with rabbit polyclonal antibody against PSaV VPg protein 72 h post infection. (D) PSaV or FCV positive cells (%) were enumerated in three independent microscope fields. All experiments were performed in triplicate and figures A and C show representative sets of results. The scale bars correspond to 20 µm. Error bars show the standard error in each sample. Asterisks indicate significant analysis, *p*<0.05.

As sialic acid moieties may by attached to glycoproteins via both *O* or *N*-linkages, the ability of PSaV to bind to LLC-PK pretreated with benzyl 4-O-β-D-galactopyranosyl-β-D-glucopyranoside (benzylGalNAc), an inhibitor of *O*-linked glycosylation, tunicamycin, an inhibitor of *N*-linked glycosylation, or PNGase F, which removes *N*-linked glycans, was examined. Pretreatment of cells with 3 mM benzylGalNAc reduced PSaV attachment to 2% of the levels observed in mock treated cells, whereas 3 µg tunicamycin ml^−1^ and 200 U PNGase F ml^−1^ pretreatment had no effect (Fig. 9A and 9B). Infection of cells by PSaV Cowden strain was also reduced by ∼97% by benzylGalNAc treatment, but was unaffected by tunicamycin or PNGase F (Fig. 9C and 9D). As a control, the FCV F9 strain, known to use *N*-linked sialic acid, was examined. As expected, FCV attachment and infection of CRFK cells was significantly inhibited by pretreatment with tunicamycin and PNGases, but not by benzylGalNAc (Fig. 9B and 9D). These data indicated that PSaV attachment occurs via sialic acid linked by *O*-linked glycosylation.

## Discussion

The lack of an efficient cell culture system of human noroviruses and sapoviruses has hampered the study of virus entry and the molecular mechanisms of virus replication. Among enteric caliciviruses, PSaV Cowden strain is the only cultivable enteric sapovirus and has been shown to replicate in a continuous cell line (LLC-PK), but only in the presence of intestinal contents from gnotobiotic pigs or bile acids as a medium supplement [6]. Although virus-host cell receptor interactions are the first step in the initiation of virus infection, the exact nature of the receptors which are recognized by the *Sapovirus* genus has not been determined. In the present study, therefore, we used the cell culture adapted PSaV Cowden strain as a model to investigate the entry strategy of an enteric sapovirus *in vitro*.

Three glycoconjugates with relevance as calicivirus receptors have been described so far; human and bovine noroviruses and Tulane virus utilize HBGAs; MNV uses sialic acid linked to ganglioside or protein in a strain-dependent manner; FCV recognizes terminal sialic acid on an *N*-linked glycoprotein [4], [5], [10], [11], [12]. Our data would indicate that PSaV does not utilize HBGAs for attachment in agreement with previous work on human sapovirus GI and GV strains that also do not appear to bind HBGAs [14]. Instead, our data Indicate that PSaV, like FCV and some MNV isolates, utilizes sialic acid as a receptor for its binding and infection [4], [11], [34].

Most sialic acids which are recognized as receptors are terminal sialic acids attached to a penultimate galactose by either α2,3-linkage or α2,6-linkage. Previous results have shown that the FCV F9 strain utilizes α2,6-linked sialic acid as a receptor [4], whereas MNV-1 strain CW3 recognizes both α2,3- and α2,6-linked sialic acids for binding and infection [11]. Our data would indicate that similarly to the MNV-1 CW3 strain [11], PSaV Cowden strain recognizes both α2,3- and α2,6-linked sialic acids which are attached to glycans as receptors.

The inability of PSaV to form stable agglutinates of RBCs from numerous species, despite the presence of α2,3 and α2,6-linked sialic acid, would suggest that PSaV also forms stabilizing interactions with the specific glycoproteins to which the sialic acids are linked. This is in agreement with our observations that protease treatment reduces virus binding (Fig. 8B) and suggests that this glycoprotein (or glycoproteins) are not expressed on the surface of RBCs. In addition, the conditions under which stable agglutination occurs, i.e., pH, ionic conditions etc., often varies from virus to virus and is dependent on the species of RBCs used [35]. Therefore, it is also possible that the lack of stable agglutination was due to suboptimal conditions used in the HA assay. The identification of specific glycoproteins involved in PSaV binding and the optimal conditions for HA activity of PSaV forms the basis of ongoing work.

The different tissue distribution of α2,3- and α2,6-linked sialic acids can strongly influence viral tissue tropism and pathogenesis [11], [25], [34], [36]. In pigs, both α2,3- and α2,6-linked sialic acids are expressed along the epithelial border as well as in goblet cells of the small and large intestines [37]. In addition, both α2,3- and α2,6-linked sialic acid receptors are distributed extensively in the major organs of pigs, including the trachea, lungs, liver, kidney, spleen, heart, skeletal muscle, and cerebrum [37]. In our previous experiments, we found that pigs orally or intra-venously infected with wild-type virulent PSaV exhibited intestinal pathology as well as viremia [29]. However, systemic infections caused by this strain were not observed, and virus was not isolated from every organ, where both α2,3- and α2,6-linked sialic acid receptors are well expressed [29]. One explanation for this that the concentration of bile acids which supports the replication of PSaV in cell culture is much higher in the proximal intestine than in the blood and extraintestinal organs [6], [29]. Therefore, it is plausible that the restriction of growth of PSaV within the small intestine is at least partially due to the requirement of a high concentration of bile acids [29]. However, we cannot rule out the possibility that some other potential co-receptors may not be present at extraintestinal sites. It has been suggested that the initial attachment of a virus to a primary receptor enriches the virus at the cell surface and primes the attached virus for interaction with secondary receptor(s) at the cell surface, which is(are) necessary for virus uptake, subsequent uncoating of the nucleic acid, and infection of the target cell [25]. One well characterized example would be the multistep entry of rotavirus into cells, where various cell receptors, including the terminal sialic acid or HBGAs, integrins, and heat shock protein Hsc70 are utilized by the outer most proteins, VP4 and VP7, of rotaviruses [38].

Among caliciviruses, FCV is known to utilize not only sialic acid, but also JAM-1 for virus entry into cells; the latter presumably aiding FCV penetration into the host cells as a co-receptor [4], [13]. In agreement with this hypothesis, cyro-EM reconstruction and biochemical studies of the FCV capsid with JAM-1 indicated that JAM-1 binding results in significant conformational changes in the capsid [39], [40], [41].

Glycosylation produces different types of glycans which are typically attached to cellular proteins and lipids [32], [37]. Different members of caliciviruses seem to utilize different linkage of glycans for their binding and entry, i.e. MNV use sialic acid bearing gangliosides (CW3 like strains) or proteins (CR3 strain) [11], [34], while FCV recognizes sialic acid bearing protein components [4]. In the present study, pretreatment of PDMP had no influence on the binding and infection of PSaV, suggesting that like FCV [4], PSaV does not utilize sialic acid bearing lipids.

Protein glycosylation encompasses *N*-glycans, *O*-glycans, and glycosaminoglycans [37]. Different types of glycans which are attached to cellular proteins are utilized by the different viruses, even within the same genus. For example, adeno-associated virus type 5 (AAV5) interacts with sialic acid on *N*-linked carbohydrates, whereas AAV4 interacts with sialic acid on *O*-linked carbohydrates, and both viruses require 2,3-linked sialic acid for binding [42]. We found that the sialic acid-bearing glycans used for PSaV binding are attached to cell surface proteins in a similar manner to FCV [4]. Unlike FCV [4], however, carbohydrate moieties linked to terminal sialic acid as a receptor for PSaV are present on an *O*-linked glycoprotein.

In conclusion, we have demonstrated that unlike noroviruses, PSaV infects cells via both α2,3- and α2,6-linked sialic acids attached to O-linked glycoproteins. This work has provided new insights into the mechanisms of sapovirus entry, and may provide additional information relevant to the identification of inhibitors of sapovirus pathogenesis.

## Materials and Methods

### Cells and viruses

LLC-PK cells, Caco2 cells and HeLa cells obtained from the American Type Culture Collection (ATCC, USA) were maintained in Eagle's minimal essential medium (EMEM) containing 10% fetal bovine serum (FBS), 100 U/ml penicillin, and 100 µg/ml streptomycin. CRFK cells and MDCK cells from ATCC was grown in Dulbecco's modified Eagle's medium (DMEM) supplemented with 5% FBS, 100 U/ml penicillin, and 100 µg/ml streptomycin. MA-104 cells from ATTCC was grown in α-MEM supplemented with 5% FBS, 100 U/ml penicillin, and 100 µg/ml streptomycin. RAW264.7 cells were grown in RPMI1640 supplemented with 5% FBS, 100 U/ml penicillin, and 100 µg/ml streptomycin. The tissue culture-adapted PSaV Cowden strain was recovered from the full-length infectious clone pCV4A, and was propagated in LLC-PK cells with the supplement of bile acid [6]. The FCV F9 strain, human influenza A virus A/Puerto Rico/8/34 (H1N1) (PR8 virus) strain, human rotavirus DS-1 strain, and CVB3 Nancy strain were purchased from the ATCC. Chicken influenza A virus A/Korea/96/96 (H9N2) (Kr96 virus) strain was obtained from Animal and Plant Quarantine Agency, Korea and MNV-1 CW1 strain was a kind gift of Dr. H.W. Virgin, Washington University School of Medicine, USA. These viruses were propagated in CRFK cells, MA-104 cells, MDCK cells, HeLa cells, or RAW264.7 cells, respectively [4], [11], [43], [44], [45].

### Reagents and antibodies

Sodium periodate (Sigma-Aldrich), trypsin (Sigma-Aldrich), chymotrypsin (Sigma-Aldrich), NANA (Fluka, USA), sialyllactose (GeneChem, Korea), galactose (Sigma-Aldrich), MAL (Sigma-Aldrich), and SNL (Sigma-Aldrich) were dissolved in PBS pH 7.2. Tunicamycin (Sigma-Aldrich) and Alexa 594 (Invitrogen, USA) were dissolved in DMSO. PDMP (Calbiochem, USA) and benzylGalNAc (Sigma-Aldrich) were dissolved in ethanol. Other reagents included ^35^[S]methionine/cysteine (PerkinElmer, USA), NA (Sigma-Aldrich), SS (Prozyme, USA), PNGase F (NEB, UK), anti-PSaV capsid monoclonal antibody [46], anti-PSaV VPg polyclonal antibody, anti-FCV capsid monoclonal antibody (Santa Cruz, USA), anti-CVB3 capsid monoclonal antibody (Millipore, IL, USA), anti-Hu/NoV/GII.4/HS194 virus-like particles (VLPs) polyclonal antibody (kind gift of Dr. L. Saif, The Ohio State University, Ohio, USA), anti-influenza virus nucleoprotein monoclonal antibody (Median Diagnostic, ChunCheon, Korea), anti-rabbit IgG-FITC antibody (Jackson Immuno Research Lab, USA), and anti-mouse IgG-FITC antibody (Santa Cruz).

### Cloning, expression and purification of VP8* of rotavirus and P particles of human norovirus

VP8* of human DS-1 rotavirus (RV) strain (P[4] genotype) and P particles of VA387 (GII-4) and VA207 (GII-9) norovirus (NV) strains were cloned, expressed and purified as described previously [26], [27]. Briefly, the cDNAs encoding RV DS-1 strain VP8* and encoding NV VA387 or VA207 P particles with cysteine peptide were cloned into the expression vector pGEX- 4T-1 (glutathione *S*-transferase [GST]–gene fusion system; GE Healthcare Life Sciences, Piscataway, NJ). After sequence confirmation, the recombinant GST-VP8 and GST-P fusion proteins were expressed in *Escherichia coli* strain BL21 as described previously (Table 2) [26], [27]. Expression of protein was induced by IPTG (isopropyl-β-D-thiogalactopyranoside; 0.2 mM) at room temperature (22°C) overnight. RV VP8-GST and NV GST-P fusion proteins were purified using the Pierce GST spin purification kit (Pierce, IL, USA) according to the manufacturer's protocol. The P particles of VA387 and VA207 were released from GST by thrombin (Sigma-Aldrich) digestion at room temperature overnight. The concentration of the purified RV VP8* and NV P particles were determined by measuring the absorbance at 280 nm.

**Table 2:** Primers used for amplification of the rotavirus VP8* and norovirus P domain with cysteine-containing tag.

Name	Sequence (5′ to 3′)	Sense	Enzyme	Construct generated
VA387-PF	GCACGGATCCTCAAGAACTAAACCATTCACC	+	BamHI	P-CDCRGDCFC
VA387-PCR	GCATGCGGCCGCTTAGCAAAAGCAATCGCCACGGCAATCGCATAATGCACGTCTGCGCCCCGC	-	NotI	
VA207-PF	GCACGAATTC TCA AAGACTAAGG CATTCACT	+	EcoRI	P-CDCRGDCFC
VA207-PCR	GCATGCGGCCGCTTAGCAAAAGCAATCGCCACGGCAATCGCATTGGATC CTTCTGCGCC CACT	-	NotI	
Wa-VP8F	GT GGATCC ATGGCTTCACTCATTTATAGAC	+	BamHI	VP8 full-length
Wa-VP8R	GC CTCGAG TCATCTAGTATTTTGAATTGGTGG	-	Xho I	

Bold letters pertain to cysteine-containing tag.

### Hemagglutination assay

Hemagglutination (HA) assay was performed using RBCs from animals and humans. Blood from pig, cow, chicken, and rat were obtained from the College of Veterinary Medicine, Chonnam National University, and human blood was provided by volunteer donors (human ABO, Lewis O^a+b-^, Lewis O^a−b+^, and Lewis O^a−b−^ types) at Chonnam National University Hospital. RBCs were packed in PBS pH 7.2 without Ca^2+^, and were centrifuged at 500×g for 10 min.

PSaV Cowden strain (10 µg/ml), human influenza virus PR8 (H1N1) (5 µg/ml starting dilutions), P particles (10 µg/ml) of norovirus VA387 strain, and VP8* of rotavirus DS-1 strain (10 µg/ml) were diluted serially 2 fold in PBS (0.01 M sodium phosphate, 0.15 M NaCl, pH 5.5) using V-shaped 96-well plates. The HA activity was tested by mixing equal volume of RBCs 1% to the prepared viruses or protein dilutions. The reactions were allowed to proceed for 1 h at 4°C or 20°C, and the agglutination of RBCs was observed and recorded [10]. The HA titer was the reciprocal of the highest virus dilution that allowed sedimentation of the RBCs compared to control wells.

### Synthetic oligosaccharides binding assay

The synthetic oligosaccharide-based histo-blood group antigen binding assay was carried out as described elsewhere [47], [48]. Briefly, 96-microtiter plates were coated with PSaV Cowden strain (10 µg/ml), P particles of NV VA207 (10 µg/ml) and VA387 (10 µg/ml) strains, human influenza virus PR8 strain (H1N1) (10 µg/ml), or VP8* of RV DS-1 strain (10 µg/ml) at 4°C overnight. Coated plates were blocked with 5% bovine serum albumin (BSA) for 1 h at room temperature, and each synthetic oligosaccharide-polyacrylamide (PAA)-biotin conjugate (10 µg/ml) was then added and further incubated at 4°C overnight. Oligosaccharides used in this study included Lewis antigens (Le^a^, Le^b^, Le^x^, and Le^y^), H type 1, H type 2, H type 3, type A disaccharide, type B disaccharide, type A trisaccharide, type B trisaccharide, sLe^a^ and sLe^x^ tetrasaccharides, which were conjugated with biotin (GlycoTech Co, USA; Table 1). Bound oligosaccharides were detected using HRP-conjugated-streptavidin (Jackson Immuno Research Lab, USA). The signal was visualized by TMB (Komabiotech, Korea) followed by measurement at 450 nm. In each step, the plates were incubated for 1 h at 37°C, after which they were washed five times with PBS-Tween 20.

### Labeling of viruses with ^35^[S] methionine/cysteine

Labeling of PSaV Cowden, FCV F9, CVB3 Nancy, MNV-1 CW1, and influenza virus Kr96 strains with ^35^[S]methionine/cysteine were carried out as described previously [4]. Briefly, confluent monolayers of permissible cells for above viruses in five 175 cm^3^ flasks were infected with above each strain, respectively, at a multiplicity of infection (MOI) of 0.1 for 4 h at 37°C. The medium was replaced with RPMI 1640 lacking methionine and cysteine (Sigma-Aldrich) for 2 h. The medium was then supplemented with 1 Mbq ^35^[S] methionine/cysteine. After 72 h (PSaV Cowden strain), 16 h (FCV F9 strain), 16 h (CVB3 Nancy strain), 16 h (MNV-1 CW1 strain), and 72 h (influenza virus Kr96 strain) post virus inoculation, the cultured virus was frozen and thawed three times. Each virus was pelleted by ultracentrifugation for 10 h at 104,000×g in a Hitachi P28S rotor, and then was purified by sucrose gradient density ultracentrifugation for 10 h at 104,000×g using a Hitachi P28S rotor.

### Labeling of viruses with Alexa 594

Labeling of PSaV Cowden, FCV F9, CVB3 Nancy, MNV-1 CW1, P domain of VA387 strain and influenza virus Kr96 strains with Alexa 594 (Invitrogen) was performed following the manufacturer's instruction. Briefly, 1 part of Alexa 594 solution was mixed with 9 parts of a solution containing 1×10^8^ pfu/ml of above each strain or 9 parts of a solution containing 500 µg/ml of P domain of VA387 strain. Each reaction was mixed thoroughly for 30 sec and was then incubated for 1 h at room temperature.

### Attachment assay with radiolabeled-viruses

Attachment assays with ^35^[S]methionine/cysteine-labeled PSaV Cowden, FCV F9, CVB3 Nancy, MNV-1 CW1 or influenza virus Kr96 strains to LLC-PK, CRFK, HeLa, RAW264.7 or MDCK cell lines were performed as described previously with slight modifications [4]. Briefly, subconfluent monolayers of permissible cells grown on 6-well plates were mixed with purified ^35^[S]methionine/cysteine-labeled above each strain (50,000 c.p.m.), and were then incubated for 45 min on ice. Cells were washed three times with ice-cold PBS, after which they were lysed with 0.1% SDS and 0.1 M NaOH. Total radioactivity in the cell lysate was determined by liquid scintillation counting [4].

### Attachment assay with Alexa 594-labeled viruses

To visualize virus attachment to subconfluent monolayers of cells grown on the confocal dish were pretreated with or without inhibitors or enzymes, as described above. Mock- or reagent-treated cells were then inoculated with PSaV Cowden, FCV F9, CVB3 Nancy, MNV-1 CW1, P domain of VA387 strain or influenza virus Kr96 strains labeled with Alexa 594 dye or Alexa 594 dye alone, after which they were incubated for 5 min on ice. Cells were washed 1 time with cold PBS 1% containing fetal bovine serum (PBS-FBS), fixed with 4% formaldehyde in cold PBS for 10 min, and washed 3 times with cold PBS. The cells which were incubated with Alexa 594 dye-labeled viruses or P domain of VA387 strain were stained with 300 nM 4′,6-diamidino-2-phenylindole (DAPI) solution for nucleus staining, mounted with using SlowFade Gold antifade reagent (Invitrogen), and examined using an EZ-C1 confocal microscope and EZ-C1 software (Nikon, Japan). Laser and microscope settings were adjusted according to the manufacturer's instructions. Cells infected with viruses unlabeled with Alexa dye were analyzed by immunofluorescence, as described below.

### Attachment assay with RT-qPCR

Attachment assays by RT-qPCR with PSaV Cowden, MNV CW1 or VSV-G protein pesudotyped lentivirus (LV) strains to LLC-PK cell line was performed as described above using Alexa of radio-labeled virus. Briefly, subconfluent monolayers of LLC-PK cells on 24-well plate were pretreated with 50 µM PDMP or 5 mM NaIO_4_. PDMP-treated cells were inoculated with above each strain (3 TCID_50_ of PSaV and MNV; 1.25 transducing units per cell of LV), and were then incubated for 45 min on ice. Cells were washed three times with ice-cold PBS, Viral RNA was immediately extracted and analyzed by RT-qPCR with primer specific to each virus. RNA was extracted using GenElute Mammalian Total RNA Miniprep Kit (Sigma). One hundred nanograms were subsequently reverse transcribed using random hexamers. Fragments of ∼200 bp were amplified using the following gene-specific primers: PSaV: 5′-CAACAATGGCACAACAACG-3′ (forward) and 5′-ACAAGCTTCTTCACCCCACA-3′ (reverse); MNV-1: 5′-TGGACAACGTGGTGAAGGAT-3′ (forward) and 5′-CAAACATCTTTCCCTTGTTC-3′ (reverse), and LV-WPRE: 5′-TCGGCCCTCAATCCAGCGGA-3′ (forward) and 5′-TCGTCTGAGGGCGAAGGCGA-3′ (reverse). Standard curves were generated for all genes quantified. Additional non-template and non-reverse transcriptase samples were routinely analyzed as negative controls. Data were collected using a ViiA 7 Real-time PCR System (Applied Biosystems).

### Virus infectivity assay

Infectivity assays of PSaV Cowden, FCV F9, CVB3 Nancy or influenza virus Kr96 strains in the permissible cells, respectively, were carried out as described previously with slight modifications [4]. Briefly, confluent monolayers of each permissible cell were treated with various inhibitors or enzymes as described below. Either mock or treated cells were infected with above each strain at an MOI of 0.1 pfu/cell, and were then incubated at 37°C for 1 h. Cells were washed three times with PBS, and were then replaced with maintenance medium. The cells were incubated for 72 h (PSaV Cowden), 8 h (FCV F9), 9 h (CVB3 Nancy) or 48 h (influenza virus Kr96) at 37°C prior to being fixed with 4% formaldehyde in PBS, and were analyzed by immunofluorescence assay as described below.

### Immunofluorescence assay

Immunofluorescence assays was performed as previously described [4]. Briefly, fixed cells in 8-well chamber slides were permeabilized by the addition of 0.2% Triton X-100, incubated for 5 min at room temperature, and then washed with PBS containing 0.1% newborn calf serum (PBS-NCS). Anti-PSaV VPg polyclonal antibody, anti-FCV capsid monoclonal antibody, anti-CVB3 capsid monoclonal antibody or anti-influenza virus nucleoprotein monoclonal antibody was then added at a dilution rate of 1∶100, 1∶200 or 1∶500, respectively. Chamber slides were incubated at 4°C overnight. Cells were washed 3 times with PBS-NCS, and FITC-conjugated secondary antibodies (diluted to 1∶100) were then added. Nuclei were stained with propidium iodide (PI), and cells were examined using confocal microscopy. A total of 700 cells, as indicated by PI or DAPI staining, were counted per condition, and were scored for PSaV VPg protein expression. After image analysis with Metamorph Premier v6.3 software (Molecular Devices, PA), the infected cells were counted as positive for viral antigen if they had a fluorescent intensity which was at least three times the fluorescent intensity of the uninfected controls. The percentage of positive cells was then normalized to that of the untreated control.

### Immunohistochemistry analysis

3-day-old piglets obtained from sows by hysterectomy were used to attain the small intestinal segments including the duodenum, jejunum and ileum. Segments were excised after sacrifice, immediately immersed in 10% buffered formalin, processed routinely for paraffin embedment, sectioned, and stained with hematoxylin for histology. For immunohistochemical studies, paraffin-embedded sections were deparaffinized, and were then rehydrated through graded alcohols into 0.1 M PBS, after which they were treated with 0.3% H_2_O_2_ to quench endogenous peroxidase. Sections were either incubated in PBS (200 µl), PSaV Cowden strain (1×10^6^ pfu/ml), P domain of VA 387 strain (10 µg/ml), NANA (160 mM, pH 7), PSaV (1×10^6^ pfu/ml) and NANA (160 mM, pH 7) mixture, P domain of VA 387 strain (10 µg/ml) and NANA (160 mM, pH 7) mixture with or without 1 mM or 10 mM NaIO_4_ pretreatment for 1 h at room temperature. Pretreated sections with NaIO_4_ were incubated for 30 min prior to the addition of virus. After washing with PBS, a monoclonal antibody against PSaV Cowden strain capsid protein or a polyclonal antibody against Hu/NoV/GII.4/HS194 VLPs was incubated with the sections at 4°C overnight. Sections were then rinsed 3 times with PBS, and incubated with biotinylated secondary antibody (Dako, USA) and peroxidase-conjugated streptavidin (Dako, USA). Reactions were developed with 3-amino-9-ethylcarbazol (AEC; Vector laboratories, USA), and followed by Mayer's hemalum solution (Merck, Germany) for counterstaining.

### Plaque reduction assay

Confluent monolayers of LLC-PK cells on 6-well plates were inoculated with PBS (200 µl), PSaV Cowden strain (1×10^5^ pfu/ml), or mixtures of PSaV (1×10^5^ plaque forming unit/ml) and various concentrations of NANA (20–160 mM, pH 7), after which they were incubated in a 5% CO_2_ incubator. After 2 h of virus adsorption, PBS and virus inocula were thoroughly discarded and washed 3 times with PBS. Overlay medium containing a 1× concentrated MEM, 10% FBS, 1.2% (w/v) avicel (FMC BioPolymer, Belgium), and 200 µM GCDCA was added to each well. Plates were incubated for 96 h in a 5% CO_2_ incubator. After incubation, inoculated cells were fixed with 20% trichloroacetic acid, and the avicel was then removed. Plaques were visualized by staining with 1% (w/v) crystal violet solution [49], [50].

### Treatment of cells with chemicals, metabolic inhibitors and enzymes

Subconfluent monolayers of LLC-PK, CRFK, HeLa, Caco2, RAW264.7 or MDCK cells grown on 6-well plates or 8-well chamber slides for confocal analysis were treated with chemicals, metabolic inhibitors, or enzymes optimized at the specific concentrations, incubation times and temperatures described in Table 3. After pretreatment, cells were washed three times with PBS, and binding and infectivity assays were carried out as described above. Mock and control treatments were performed at the same time.

**Table 3:** Chemicals, metabolic inhibitors, and enzymes used in this study.

Chemicals	Optimum concentration	Incubation time, temperature
Sodium periodate (NaIO_4_)	1 or 5 mM	30 min, 4°C
Neuraminidase	200 mU	1 h, 37°C
Sialidase S	40 mU	1 h, 37°C
*Maakia amurensis* lectin (MAL)	400 µg/ml	1 h, 4°C
*Sambucus nigra* lectin (SNL)	400 µg/ml	1 h, 4°C
MAL and SNL mixture	400 µg/ml each	1 h, 4°C
Trypsin	10 µg/ml	30 min, 37°C
Chymotrypsin	10 µg/ml	30 min, 37°C
PDMP	50 µM	3 d, 37°C
BenzylGalNAc	3 mM	3 d, 37°C
Tunicamycin	3 µg/ml	24 h, 37°C
PNGase F	200 U	1 h, 37°C
*N*-acetyl neuraminic acid (NANA)	20, 40, 80, 160 mM	1 h, 4°C

### Statistical analysis

Statistical analysis was performed using SPSS version 11.5.1 for window (SPSS, USA). One way analysis of variance (ANOVA) test were used. A P-value <0.05 was considered statistically significant.

### Ethics statement

All animals were handled in strict accordance with good animal practices, as described in the NIH Guide for the Care and Use of Laboratory Animals (NIH Publication No. 85–23, 1985, revised 1996). The protocol was approved by the Committee on Ethics of Animal Experiments, CNU with permit number (CNU No. 2012-87). The human blood samples collected with written consent from the patients were handled in strict accordance with human subjects, as described in the Guidance for the Care and Use of Human Samples of the CNU adhered from the WMA Declaration of Helsinki (Ethical Principles for Medical Research Involving Human Subjects). The protocol was approved by the Committee for Research Ethics Concerning Human Subjects, CNU with permit number (CNU IBR No. 1040198-130807-BR-002-01).

## Supporting Information

Figure S1 **NaIO_4_ treatment does not affect binding of viruses that do not utilize carbohydrate receptors.** (A) Alexa 594 or Alexa 594-labeled CVB3 (MOI of 100 pfu/cell) were bound to HeLa cells pretreated with 1 mM or 5 mM NaIO_4_ to remove carbohydrate moieties and were subsequently examined for bound virus by confocal microscopy. (B) CVB3 (MOI of 0.1 pfu/cell) was inoculated to NaIO_4_ pretreated HeLa cells, and was subsequently analyzed by immunofluorescence assay to detect the viral capsid VP1 protein, using a mouse monoclonal antibody 9 h post infection. (C) LLC-PK cells were pretreated with 5 mM NaIO_4_ as described in the Materials and Methods section. Mock and treated cells were then incubated with PSaV or VSV-G protein pseudotyped lentivirus (LV) at a MOI of 3 TCID_50_ (PSaV) or 1.25 transducing units per cell (LV). Unbound virus was removed by washing. Viral RNA was immediately extracted and analyzed by RT-qPCR. Graphs show the mean and standard deviation of one experiment performed in biological triplicate. The scale bars correspond to 20 µm. ns: no significance; **p<0.005.
(TIF)

Figure S2 ***N*** **-acetyl neuraminic acid (NANA) reduces procine sapovirus plaque formation.** The ability of PSaV to bind to a soluble sialic acid, NANA, was analyzed by plaque reduction assay, as described in Materials and Methods. Plaque reduction assay was performed after pre-incubation of PSaV (1×10^5^ pfu/ml) with PBS or various concentrations of NANA, as indicated. The experiment was performed in triplicate and one representative set of results is shown.
(TIF)

Figure S3 **Attachment of the P domain of human norovirus VA387 to porcine intestinal sections is not blocked by** ***N*** **-acetyl neuraminic acid (NANA).** The ability of P domain of human norovirus VA387 to bind to porcine intestinal tissue sections from the duodenum was analyzed by immunohistochemistry as described in the Materials and Methods section. Tissue sections were incubated without (A) or with P domain of VA387 strain (B), pretreated with 1 mM NaIO_4_ (C) or 10 mM NaIO_4_ (D) prior to the addition of P domain of VA387 strain, or incubated with a mixture of P domain and 160 mM NANA (pH 7) (E). Binding of P domain to cells was identified by guinea-pig anti-Hu/NoV/GII.4/HS194 VLPs polyclonal antibody using immunohistochemistry and positive binding is indicated by a red/brown color. Scale bars correspond to 200 µm. This experiment was repeated three independent times and one representative set of results is shown.
(TIF)

Figure S4 **Avian influenza virus Kr96 (H9N2) requires α2,3-linked terminal sialic acids.** MDCK cells were pretreated with *V. cholerae* neuraminidase (NA; removes α2,3-, α2,6- and α2,8-linked sialic acid) or sialidase S (SS; removes α2,3-linked sialic acid) from *Streptococcus pneumoniae* at the indicated concentrations. (A) Cells were incubated with either Alex 594 alone or Alexa 594-labeled Kr96 (MOI of 100 pfu/cell), and bound virus was detected by confocal microscopy. (B) Kr96 nucleoprotein-positive cells (%) were enumerated in three independent microscope fields. All experiments were performed independently three times and figures A and B show a single representative set of results. The scale bars correspond to 20 µm.
(TIF)

Figure S5 **Human norovirus GII.4 VA387 strain does not require sialic acid as a receptor.** Caco2 cells were treated with *V. cholerae* neuraminidase (NA; removes α2,3-, α2,6- and α2,8-linked sialic acid) or sialidase S (SS; removes α2,3-linked sialic acid) from *Streptococcus pneumoniae* at the indicated concentrations. Cells were incubated with either Alexa 594 alone or Alexa 594-labeled P domain of human norovirus VA387 strain, and bound P domains were detected by confocal microscopy. This experiment was performed independently three times and the figure shows a single representative set of results. The scale bars correspond to 20 µm.
(TIF)

Figure S6 **Murine norovirus MNV-1 interacts with sialic acid on glycolipids.** RAW264.7 cells were pre-incubated with trypsin, chymotrypsin or PDMP (lipid metabolic inhibitor) at the indicated concentrations to examine which glycan moieties sialic acid is attached to. Alexa 594 alone or Alexa 594-labeled MNV-1 CW1 strain (MOI of 100) were bound to pretreated cells, and were observed for their binding activity by confocal microscopy. All experiments were performed in triplicate and figure A shows one representative sets of results. The scale bars correspond to 20 µm.
(TIF)

Figure S7 **PDMP inhibition of glycolipid synthesis reduces the binding of MNV-1 and VSV-G protein pseudotyped lentivirus without affecting porcine sapovirus.** LLCPK-1 cells were pretreated with 50 µM PDMP as described in the Materials and Methods section. Mock and treated cells were then incubated with PSaV (panel A), MNV-1 (panel B) and VSV-G protein pseudotyped lentivirus (LV, panel C) at a MOI of 3 TCID_50_ (PSaV and MNV) or 1.25 transducing units per cell (LV). Unbound virus was removed by washing. Viral RNA was immediately extracted and analyzed by RT-qPCR. Graphs show the mean and standard deviation of one of two experiments, each performed using biological triplicates. **p<0.005.
(TIF)
