# Correction: Both α2,3- and α2,6-Linked Sialic Acids on O-Linked Glycoproteins Act as Functional Receptors for Porcine Sapovirus

**DOI:** 10.1371/journal.ppat.1004267

**Published:** 2014-06-24

**Authors:** The PLOS Pathogens Staff

The Funding statement in the published article contains errors in the first sentence. Please see the correct Funding statement below.

This study was supported by Basic Science Research Program through the National Research Foundation of Korea (NRF) funded by the Ministry of Science, ICT and Future Planning (2014R1A2A2A01004292), and by a KBSI grant (C33730). IG is supported by funding from the Wellcome Trust (Ref:WT097997MA). IG is a Wellcome Senior Fellow. The funders had no role in study design, data collection and analysis, decision to publish, or presentation of the manuscript.
