# Supplementary figures and images for: Both α2,3- and α2,6-Linked Sialic Acids on O-Linked Glycoproteins Act as Functional Receptors for Porcine Sapovirus
Source: PLoS Pathog. 2014 Jun 5;10(6):e1004172. doi: 10.1371/journal.ppat.1004172 (PMC4047124; doi:10.1371/journal.ppat.1004172)

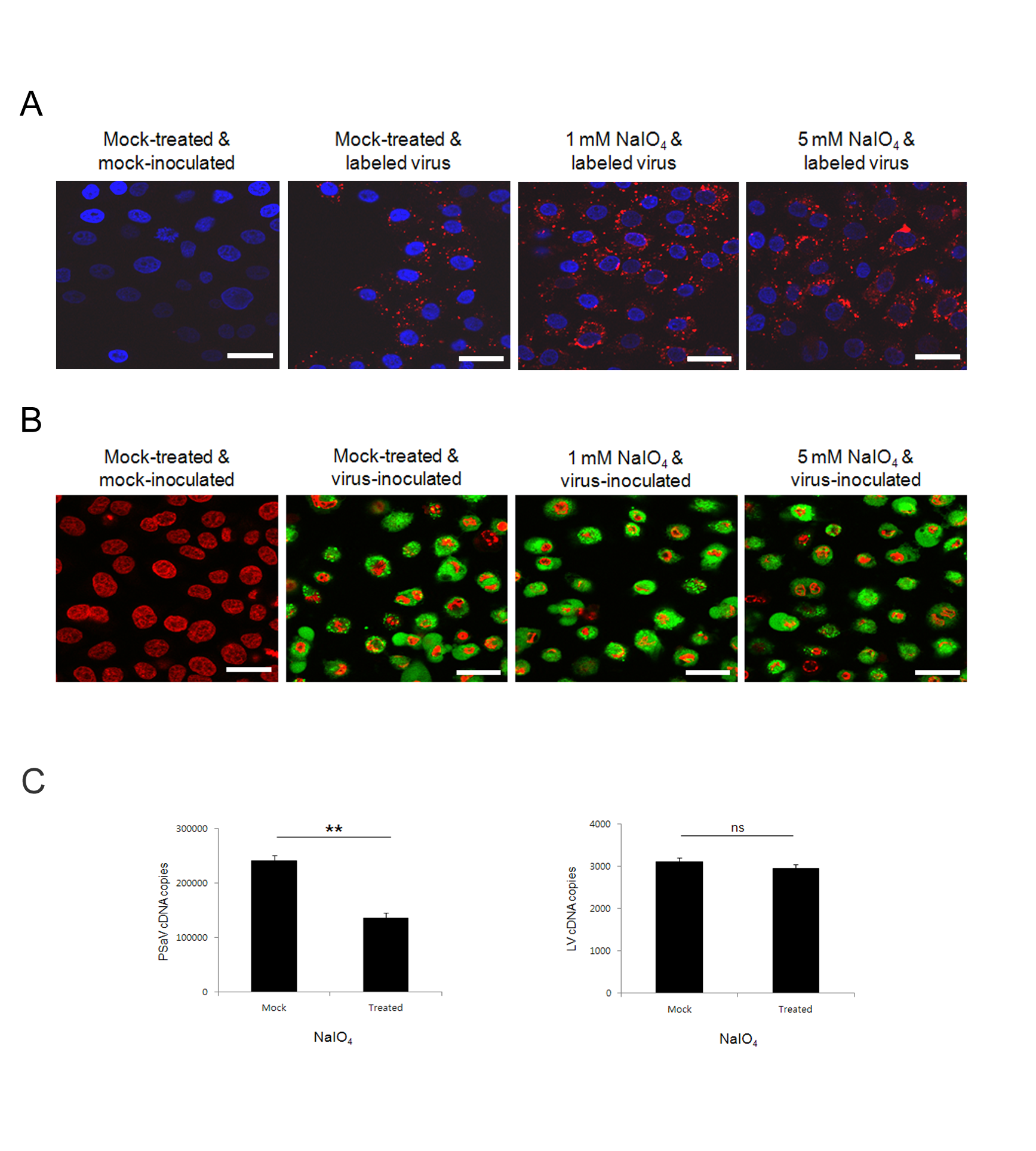

Supplement: Figure S1 — NaIO4 treatment does not affect binding of viruses that do not utilize carbohydrate receptors. (A) Alexa 594 or Alexa 594-labeled CVB3 (MOI of 100 pfu/cell) were bound to HeLa cells pretreated with 1 mM or 5 mM NaIO4 to remove carbohydrate moieties and were subsequently examined for bound virus by confocal microscopy. (B) CVB3 (MOI of 0.1 pfu/cell) was inoculated to NaIO4 pretreated HeLa cells, and was subsequently analyzed by immunofluorescence assay to detect the viral capsid VP1 protein, using a mouse monoclonal antibody 9 h post infection. (C) LLC-PK cells were pretreated with 5 mM NaIO4 as described in the Materials and Methods section. Mock and treated cells were then incubated with PSaV or VSV-G protein pseudotyped lentivirus (LV) at a MOI of 3 TCID50 (PSaV) or 1.25 transducing units per cell (LV). Unbound virus was removed by washing. Viral RNA was immediately extracted and analyzed by RT-qPCR. Graphs show the mean and standard deviation of one experiment performed in biological triplicate. The scale bars correspond to 20 µm. ns: no significance; **p<0.005. (TIF) [file ppat.1004172.s001.tif]

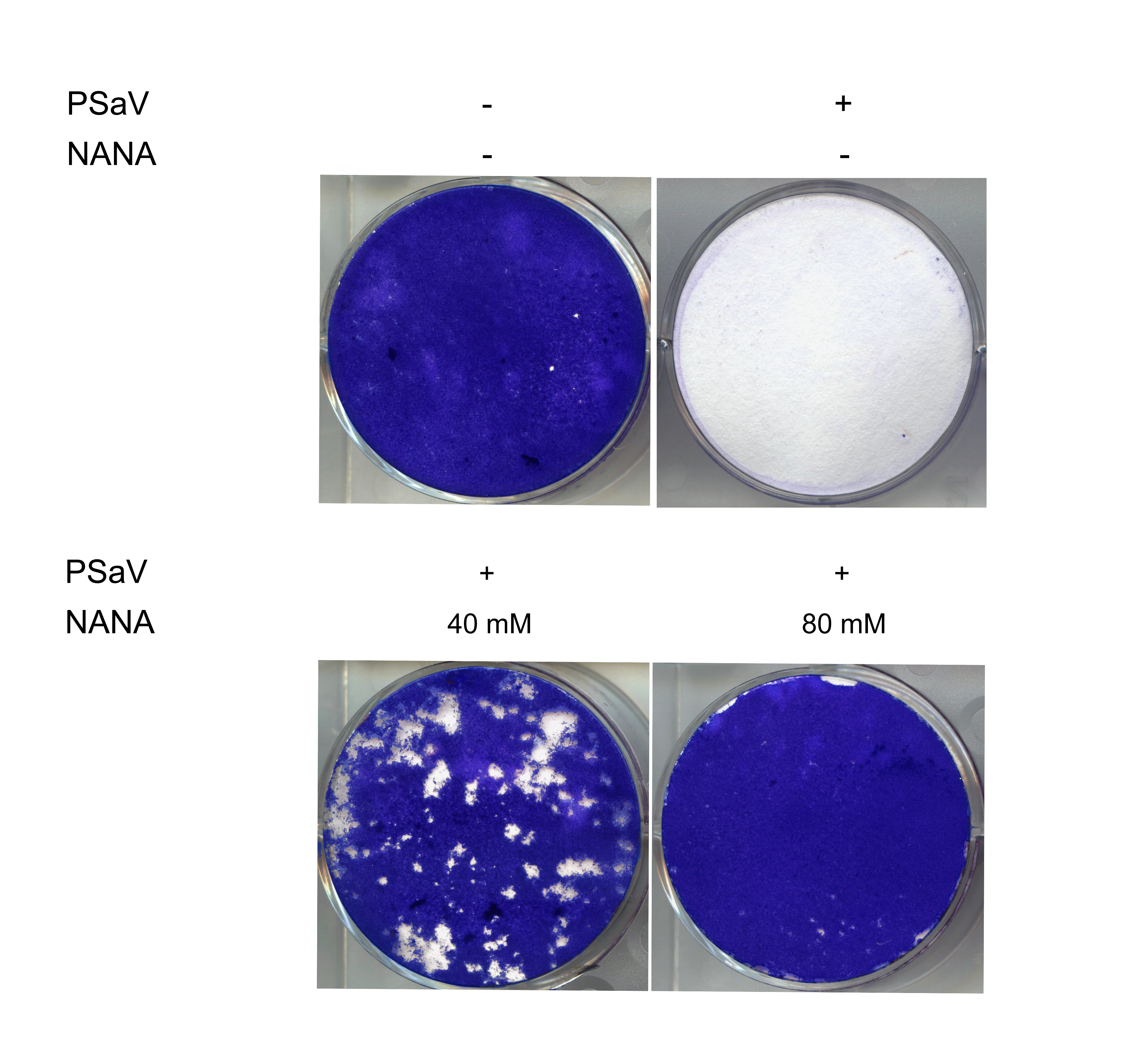

Supplement: Figure S2 — N -acetyl neuraminic acid (NANA) reduces procine sapovirus plaque formation. The ability of PSaV to bind to a soluble sialic acid, NANA, was analyzed by plaque reduction assay, as described in Materials and Methods. Plaque reduction assay was performed after pre-incubation of PSaV (1×10^5^ pfu/ml) with PBS or various concentrations of NANA, as indicated. The experiment was performed in triplicate and one representative set of results is shown. (TIF) [file ppat.1004172.s002.tif]

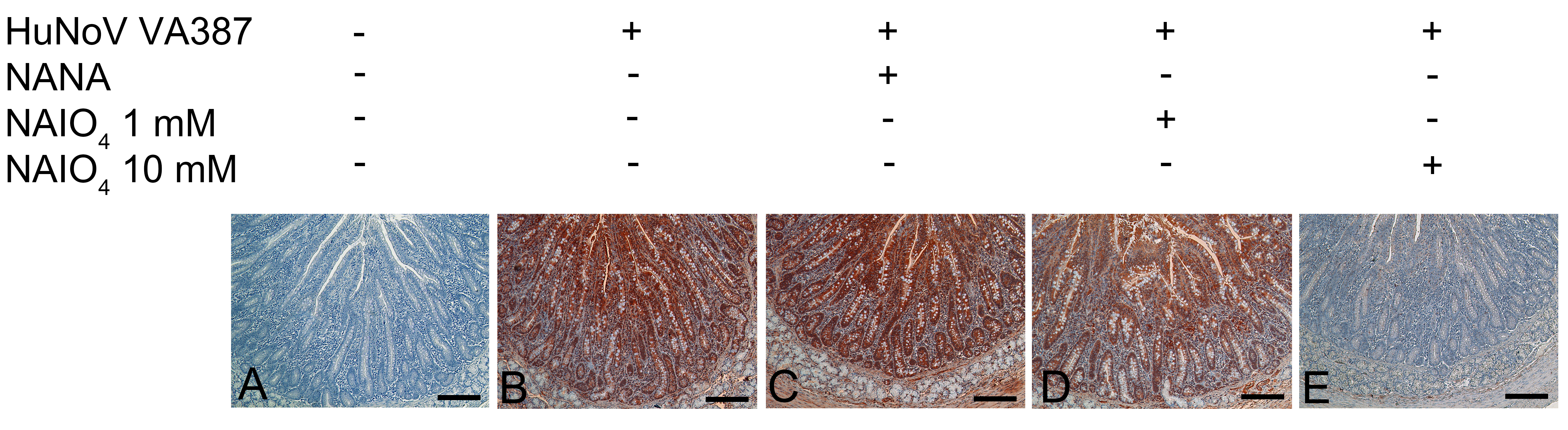

Supplement: Figure S3 — Attachment of the P domain of human norovirus VA387 to porcine intestinal sections is not blocked by N -acetyl neuraminic acid (NANA). The ability of P domain of human norovirus VA387 to bind to porcine intestinal tissue sections from the duodenum was analyzed by immunohistochemistry as described in the Materials and Methods section. Tissue sections were incubated without (A) or with P domain of VA387 strain (B), pretreated with 1 mM NaIO4 (C) or 10 mM NaIO4 (D) prior to the addition of P domain of VA387 strain, or incubated with a mixture of P domain and 160 mM NANA (pH 7) (E). Binding of P domain to cells was identified by guinea-pig anti-Hu/NoV/GII.4/HS194 VLPs polyclonal antibody using immunohistochemistry and positive binding is indicated by a red/brown color. Scale bars correspond to 200 µm. This experiment was repeated three independent times and one representative set of results is shown. (TIF) [file ppat.1004172.s003.tif]

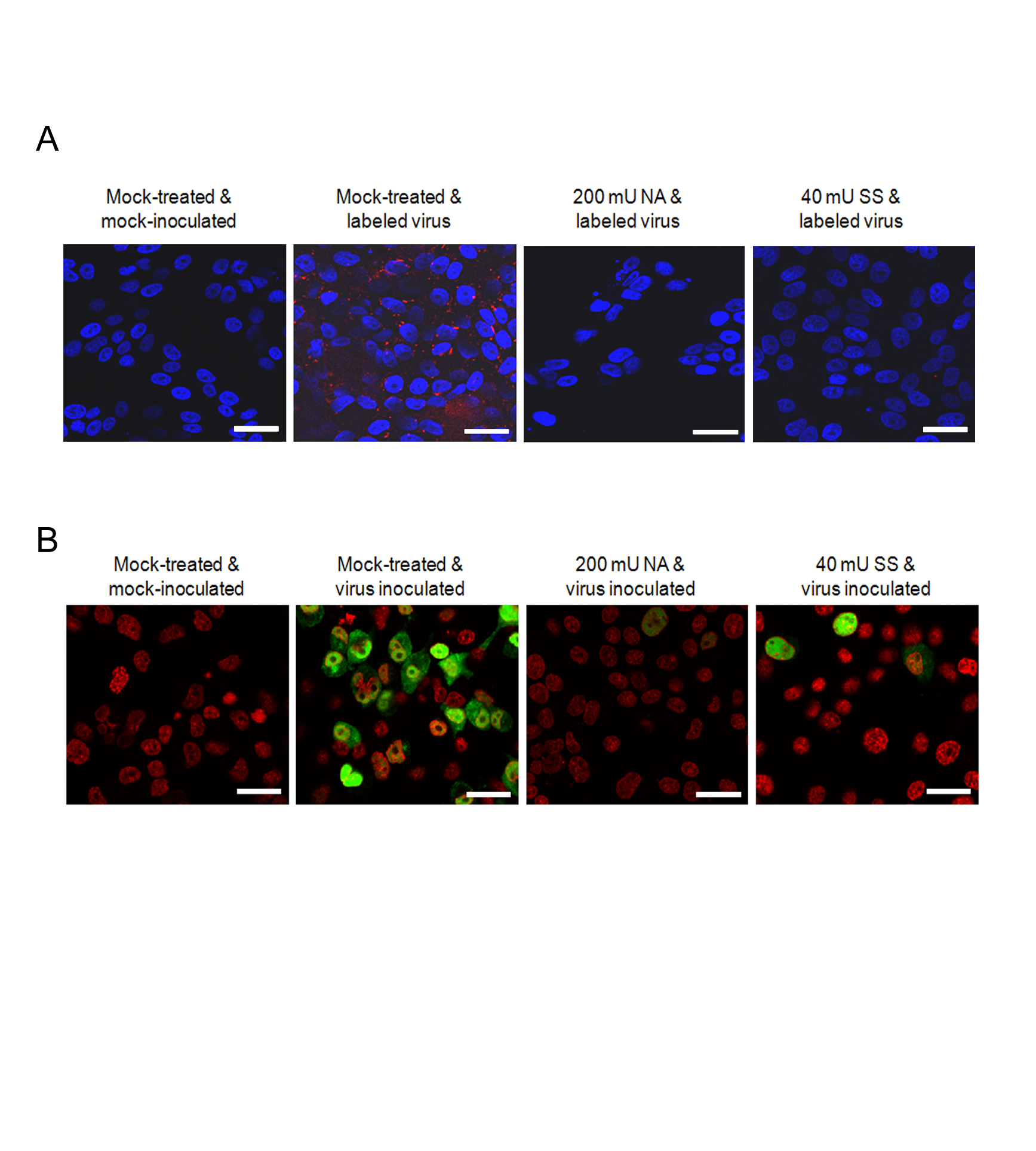

Supplement: Figure S4 — Avian influenza virus Kr96 (H9N2) requires α2,3-linked terminal sialic acids. MDCK cells were pretreated with V. cholerae neuraminidase (NA; removes α2,3-, α2,6- and α2,8-linked sialic acid) or sialidase S (SS; removes α2,3-linked sialic acid) from Streptococcus pneumoniae at the indicated concentrations. (A) Cells were incubated with either Alex 594 alone or Alexa 594-labeled Kr96 (MOI of 100 pfu/cell), and bound virus was detected by confocal microscopy. (B) Kr96 nucleoprotein-positive cells (%) were enumerated in three independent microscope fields. All experiments were performed independently three times and figures A and B show a single representative set of results. The scale bars correspond to 20 µm. (TIF) [file ppat.1004172.s004.tif]

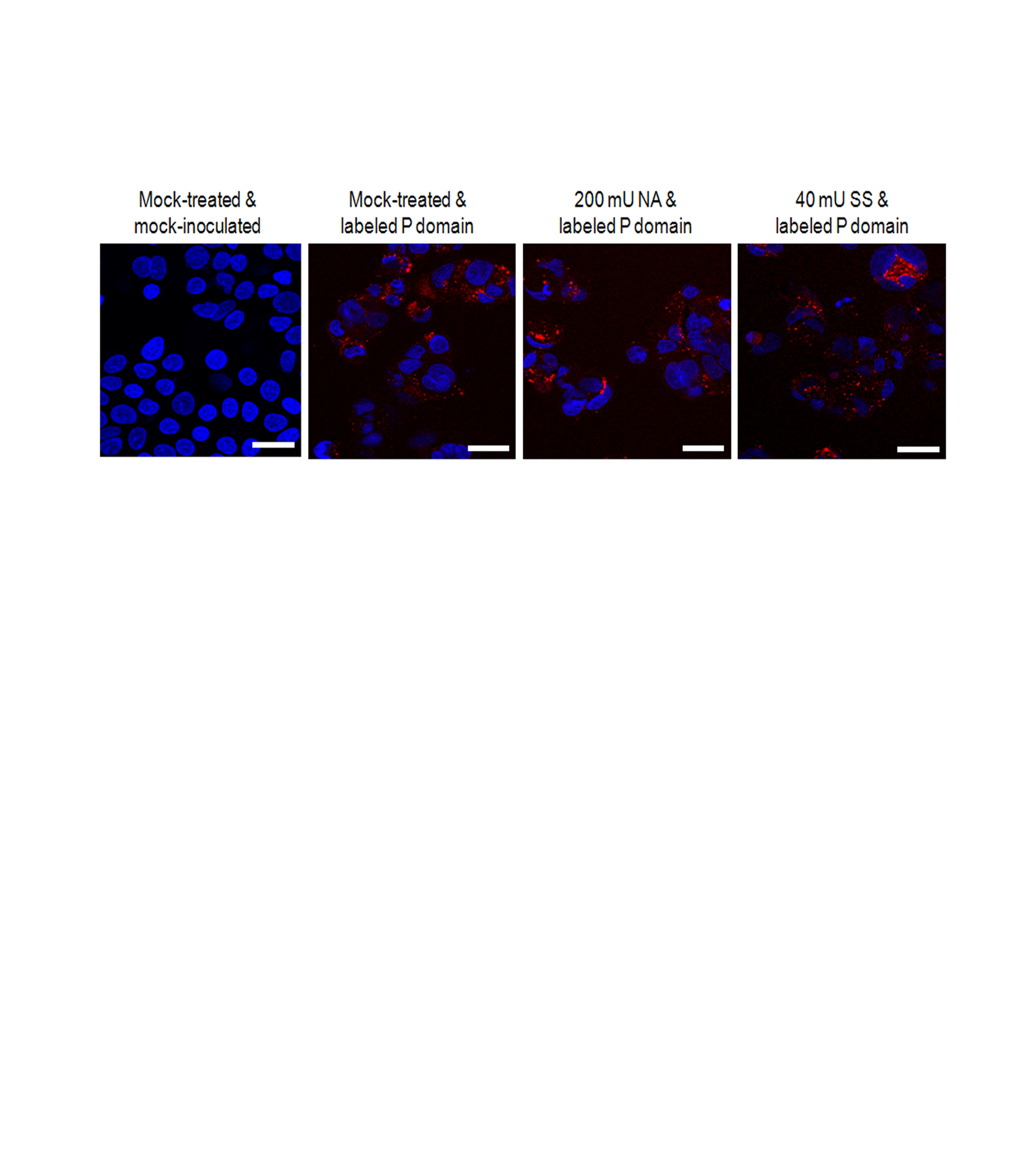

Supplement: Figure S5 — Human norovirus GII.4 VA387 strain does not require sialic acid as a receptor. Caco2 cells were treated with V. cholerae neuraminidase (NA; removes α2,3-, α2,6- and α2,8-linked sialic acid) or sialidase S (SS; removes α2,3-linked sialic acid) from Streptococcus pneumoniae at the indicated concentrations. Cells were incubated with either Alexa 594 alone or Alexa 594-labeled P domain of human norovirus VA387 strain, and bound P domains were detected by confocal microscopy. This experiment was performed independently three times and the figure shows a single representative set of results. The scale bars correspond to 20 µm. (TIF) [file ppat.1004172.s005.tif]

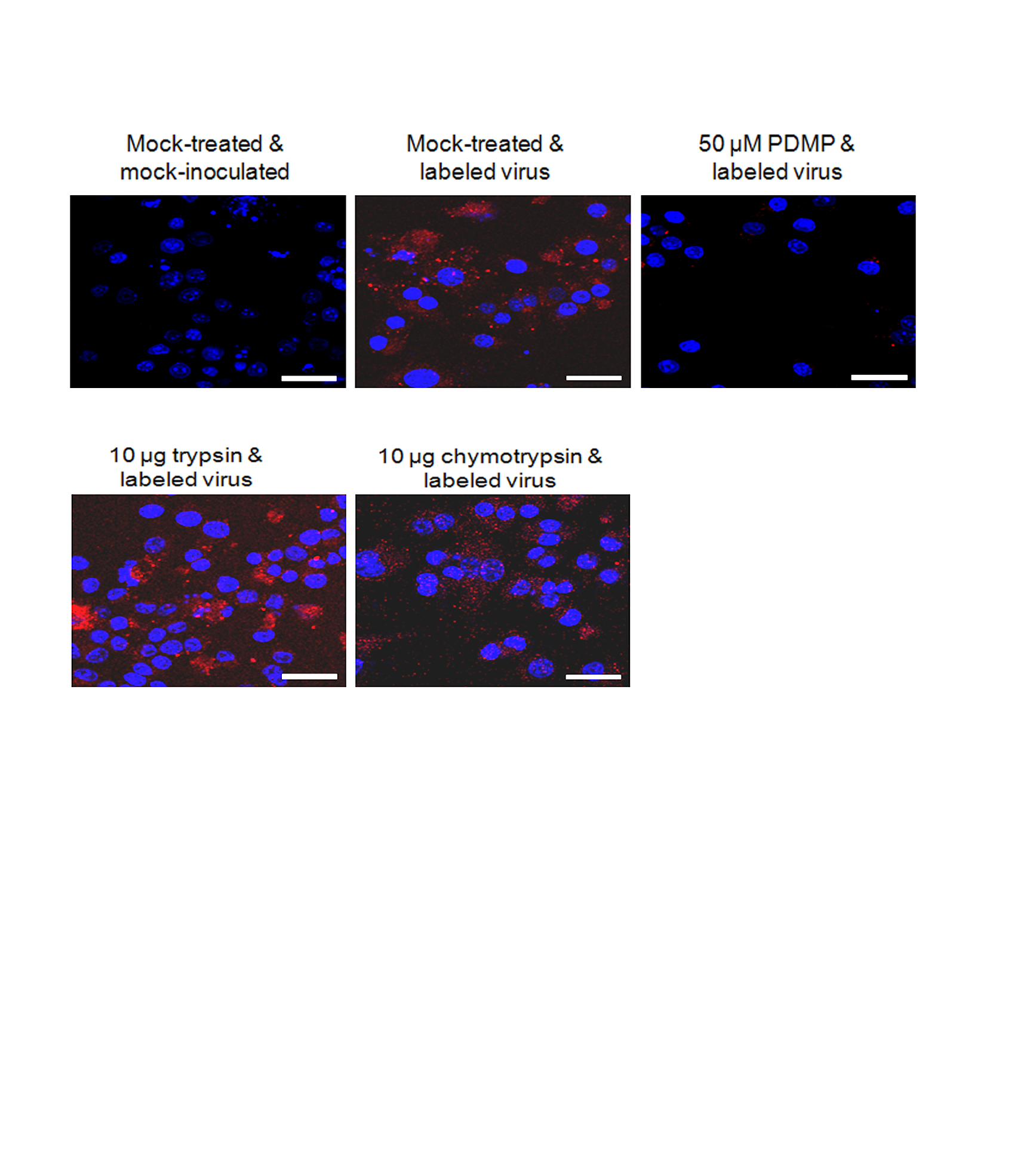

Supplement: Figure S6 — Murine norovirus MNV-1 interacts with sialic acid on glycolipids. RAW264.7 cells were pre-incubated with trypsin, chymotrypsin or PDMP (lipid metabolic inhibitor) at the indicated concentrations to examine which glycan moieties sialic acid is attached to. Alexa 594 alone or Alexa 594-labeled MNV-1 CW1 strain (MOI of 100) were bound to pretreated cells, and were observed for their binding activity by confocal microscopy. All experiments were performed in triplicate and figure A shows one representative sets of results. The scale bars correspond to 20 µm. (TIF) [file ppat.1004172.s006.tif]

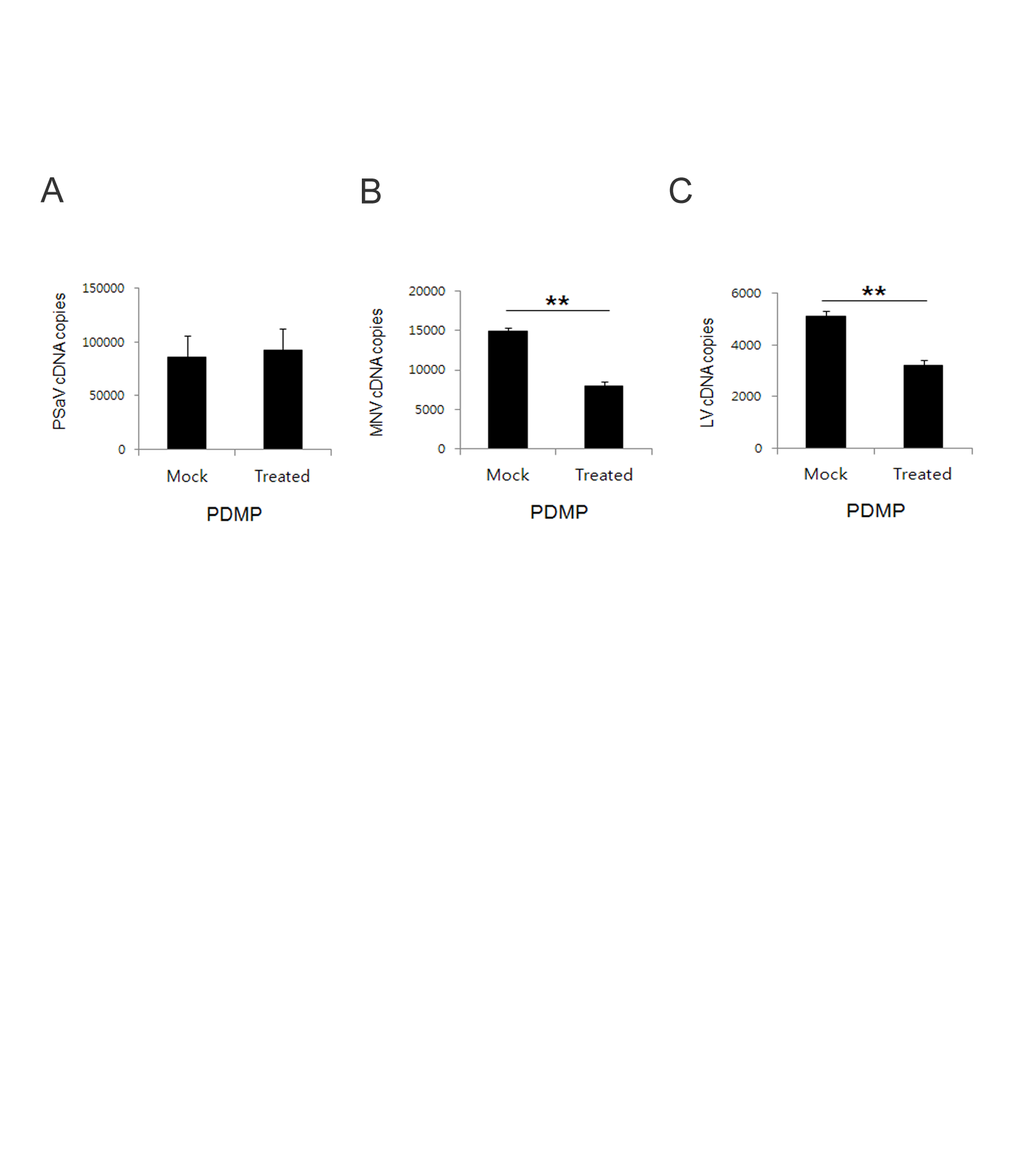

Supplement: Figure S7 — PDMP inhibition of glycolipid synthesis reduces the binding of MNV-1 and VSV-G protein pseudotyped lentivirus without affecting porcine sapovirus. LLCPK-1 cells were pretreated with 50 µM PDMP as described in the Materials and Methods section. Mock and treated cells were then incubated with PSaV (panel A), MNV-1 (panel B) and VSV-G protein pseudotyped lentivirus (LV, panel C) at a MOI of 3 TCID50 (PSaV and MNV) or 1.25 transducing units per cell (LV). Unbound virus was removed by washing. Viral RNA was immediately extracted and analyzed by RT-qPCR. Graphs show the mean and standard deviation of one of two experiments, each performed using biological triplicates. **p<0.005. (TIF) [file ppat.1004172.s007.tif]
